# Efficacy of RTS,S/AS01_E_ malaria vaccine administered according to different full, fractional, and delayed third or early fourth dose regimens in children aged 5–17 months in Ghana and Kenya: an open-label, phase 2b, randomised controlled trial

**DOI:** 10.1016/S1473-3099(22)00273-0

**Published:** 2022-09

**Authors:** Aaron M Samuels, Daniel Ansong, Simon K Kariuki, Samuel Adjei, Anne Bollaerts, Christian Ockenhouse, Nelli Westercamp, Cynthia K Lee, Lode Schuerman, Dennis K Bii, Lawrence Osei-Tutu, Martina Oneko, Marc Lievens, Maame Anima Attobrah Sarfo, Cecilia Atieno, Danielle Morelle, Ashura Bakari, Tony Sang, Erik Jongert, Maame Fremah Kotoh-Mortty, Kephas Otieno, François Roman, Patrick Boakye Yiadom Buabeng, Yaw Ntiamoah, Opokua Ofori-Anyinam, Tsiri Agbenyega, RTS,S study group, David Sambian, Albert Agordo Dornudo, Lydia Nana Badu, Kwame Akoi, Evans Antwi, Kelvin Onoka, Kevin K'Orimba, Paul Ndaya Oloo, Elizabeth Leakey, Emilia Gvozdenovic, Cristina Cravcenco, Pascale Vandoolaeghe, Johan Vekemans, Karen Ivinson

**Affiliations:** a Malaria Branch, Division of Parasitic Diseases and Malaria, Center for Global Health, United States Centers for Disease Control and Prevention, Kisumu, Kenya; b Malaria Branch, Division of Parasitic Diseases and Malaria, Center for Global Health, Centers for Disease Control and Prevention, Atlanta, GA, USA; c Kwame Nkrumah University of Science and Technology/Agogo Presbyterian Hospital, Agogo, Asante Akyem, Ghana; d Centre for Global Health Research, Kenya Medical Research Institute, Kisumu, Kenya; e GSK, Wavre, Belgium; f PATH's Malaria Vaccine Initiative, Washington, DC, USA

## Abstract

**Background:**

Controlled infection studies in malaria-naive adults suggest increased vaccine efficacy for fractional-dose versus full-dose regimens of RTS,S/AS01. We report first results of an ongoing trial assessing different fractional-dose regimens in children, in natural exposure settings.

**Methods:**

This open-label, phase 2b, randomised controlled trial is conducted at the Malaria Research Center, Agogo, Ashanti Region (Ghana), and the Kenya Medical Research Institute and the US Centers for Disease Control and Prevention site in Siaya County (Kenya). We enrolled children aged 5–17 months without serious acute or chronic illness who had previously received three doses of diphtheria, tetanus, pertussis, and hepatitis B vaccine and at least three doses of oral polio vaccine. Children were randomly assigned (1:1:1:1:1) using a web-based randomisation system with a minimisation procedure accounting for centre to receive rabies control vaccine (M012 schedule) or two full doses of RTS,S/AS01_E_ at month 0 and month 1, followed by either full doses at months 2 and 20 (group R012-20 [standard regimen]), full doses at months 2, 14, 26, and 38 (R012-14), fractional doses at months 2, 14, 26, and 38 (Fx012-14), or fractional doses at months 7, 20, and 32 (Fx017-20). The fractional doses were administered as one fifth (0·1 mL) of the full RTS,S dose (0·5 mL) after reconstitution. All vaccines were administered by intramuscular injection in the left deltoid. The primary outcome was occurrence of clinical malaria cases from month 2·5 until month 14 for the Fx012-14 group versus the pooled R012-14 and R012-20 groups in the per-protocol set. We assessed incremental vaccine efficacy of the Fx012-14 group versus the pooled R012-14 and R012-20 group over 12 months after dose three. Safety was assessed in all children who received at least one vaccine dose. This trial is registered with ClinicalTrials.gov, NCT03276962.

**Findings:**

Between Sept 28, 2017, and Sept 25, 2018, 2157 children were enrolled, of whom 1609 were randomly assigned to a treatment group (322 to each RTS,S/AS01_E_ group and 321 to the rabies vaccine control group). 1500 children received at least one study vaccine dose and the per-protocol set comprised 1332 children. Over 12 months after dose three, the incremental vaccine efficacy in the Fx012-14 group versus the pooled R012-14 and R12-20 groups was −21% (95% CI −57 to 7; p=0·15). Up to month 21, serious adverse events occurred in 48 (16%) of 298 children in the R012-20 group, 45 (15%) of 294 in the R012-14 group, 47 (15%) of 304 in the Fx012-14 group, 62 (20%) of 311 in the Fx017-20 group, and 71 (24%) of 293 in the control group, with no safety signals observed.

**Interpretation:**

The Fx012-14 regimen was not superior to the standard regimen over 12 months after dose three. All RTS,S/AS01_E_ regimens provided substantial, similar protection against clinical malaria, suggesting potential flexibility in the recommended dosing regimen and schedule. This, and the effect of annual boosters, will be further evaluated through 50 months of follow-up.

**Funding:**

GlaxoSmithKline Biologicals; PATH's Malaria Vaccine Initiative.

## Introduction

The unprecedented deployment of malaria interventions since 2000 led to a considerable decrease in malaria morbidity and mortality. However, progress in malaria control has stagnated in recent years. WHO estimated that 241 million cases of malaria occurred in 2020, resulting in 627 000 deaths, of which 77% were in children younger than 5 years and approximately 95% occurred in the WHO African region.^1^ The efficacy of existing preventive interventions and life-saving therapies is threatened by the development of insecticide resistance by the vector and drug resistance by the parasite, and thus new tools are needed.

**Research in context**

**Evidence before this study**

We did a systematic literature search of PubMed on Nov 12, 2021, for any clinical trial of the RTS,S/AS01 malaria vaccine with a fractional dose, using the search string ((“RTS,S” [All Fields]) AND (“fractional” [All Fields])) and clinical trial (article type). We restricted the search to publications in English. Of the four manuscripts identified, three reported results of phase 2a controlled human malaria infection (CHMI) studies assessing vaccine efficacy in adults, and one evaluated the effect of a change in the vaccine regimen on the quality of antibody responses.

**Added value of this study**

To our knowledge, this is the first study to evaluate proof-of-concept for a fractional-dose regimen of RTS,S/AS01_E_ in children aged 5–17 months, under conditions of natural exposure. We evaluated whether immunisation regimens with a delayed fractional third dose or an early fractional fourth dose are efficacious or increase the protective efficacy and effect of RTS,S/AS01_E_ against malaria in countries with moderate-to-high malaria endemicity. We additionally evaluated whether timing of the dose administration affects vacine efficacy and provides new information on the immunogenicity and safety of two fractional-dose regimens of RTS,S/AS01_E_ in children. Vaccine efficacy against clinical malaria of a fractional regimen (full doses at 0 and 1 month and one fifth of a full dose at month 2) was not superior to that of the full-dose schedule over 12 months after the first three doses. Improved vaccine efficacy was not observed for the delayed fractional-dose (third, fractional dose at month 7) regimen either. However, we observed a similar vaccine efficacy for all RTS,S/AS01_E_ groups. All regimens were immunogenic and well tolerated.

**Implications of all the available evidence**

The phase 2a CHMI studies in malaria-naive adults showed that regimens containing a delayed third or early fourth fractional dose of RTS,S/AS01_E_ confer high protection against clinical malaria. Although the primary endpoint or our study was not met, our results suggest that the use of a fractional RTS,S/AS01_E_ regimen (with some flexibility around the time of administration of the third or fourth doses) provides protection against clinical malaria similar to the standard month 0, 1, and 2 full-dose regimen, over an initial 20-month period of follow-up from first vaccination. If these findings are confirmed up to 50 months of follow-up, they might confer large public health benefit, as a fractional dose regimen could potentially allow vaccination of more children with the same amount of vaccine as compared with a full dose.

RTS,S/AS01_E_ (hereafter referred to as RTS,S; GSK, Wavre, Belgium) is the only vaccine currently recommended against malaria. A phase 3 clinical trial was conducted at 11 sites across seven sub-Saharan Africa countries between 2009 and 2014. Vaccine efficacy against all episodes of clinical malaria in children aged 5–17 months when administered according to a 0, 1, and 2 month primary schedule (M012) was 55·1% (95% CI 50·5 to 59·3) over 12 months of follow-up.^2^ Over 4 years of follow-up, vaccine efficacy against clinical malaria was 28·3% (95% CI 23·3 to 32·9) and against severe malaria was 1·1% (−23·0 to 20·5); the addition of a fourth dose at month 20 (M012-20) increased vaccine efficacy against clinical malaria to 36·3% (31·8 to 40·5) and against severe malaria to 32·2% (13·7 to 46·9).^3^ These results supported pilot implementation of RTS,S through the Expanded Programmes on Immunisation in Ghana, Kenya, and Malawi.^4^ On Oct 6, 2021, WHO recommended widespread use of RTS,S in children in sub-Saharan Africa and areas with moderate-to-high *Plasmodium falciparum* transmission.^5^

Concomitantly, efforts continue to improve vaccine efficacy, durability of protection, and availability of RTS,S. Several controlled human malaria infection (CHMI) studies in malaria-naive adults have suggested that a regimen containing a fractional dose of different RTS,S formulations confers high protection against *P falciparum* infection.^6^, ^7^, ^8^, ^9^ One CHMI study found a vaccine efficacy of 86·7% (95% CI 66·8–94·6) with a delayed third fractional vaccine dose (one fifth of the full dose) at month 7 (M017) compared with 62·5% (29·4–80·1) with the standard M012 full-dose regimen, at month 8 after CHMI.^7^ These findings could result in substantial public health impact if corroborated in children in the setting of natural exposure, because a regimen with a fractional third or fourth dose might result in more children being vaccinated with RTS,S than when using a standard full-dose regimen. This has the potential to improve population-level vaccine availability, and thus reduce malaria morbidity and mortality.

We report results up to 20 months of follow-up of an ongoing phase 2b trial that aims to establish proof-of-concept for the use of fractional-dose regimens of RTS,S in children aged 5–17 months at first vaccination, under conditions of natural exposure. Therefore, we compared a fractional-dose regimen to the currently recommended standard full-dose schedule (M012). We also explored the effect of a delayed third dose in a fractional-dose regimen (M017), and the effect of an early full or fractional fourth dose (at month 14) on vaccine efficacy, over 1 year of follow-up after dose three and up to month 20. Immunogenicity and safety of all doses were also assessed up to month 21.

## Methods

### Study design and participants

This open-label, phase 2b, randomised controlled trial is conducted by the Malaria Research Center, Agogo, Ashanti Region (Ghana), and the Kenya Medical Research Institute and the US Centers for Disease Control and Prevention (KEMRI/CDC) site in Siaya County (Kenya). Both sites have perennial, moderate-to-high malaria transmission. *P falciparum* prevalence by microscopy was 17% in 2016 in the Ashanti Region and 39% in 2015 at the Kenya trial site; reported use of long-lasting insecticidal nets the night before the survey was 51% in the Ashanti Region and 91% at the Kenya site among children younger than 5 years.^10^, ^11^

We enrolled children without serious acute or chronic illness aged 5–17 months if they had previously received three doses of diphtheria, tetanus, pertussis, and hepatitis B vaccine and at least three doses of oral polio vaccine. Recruitment procedures and full inclusion and exclusion criteria are described in appendix pp 1–2. The families of all screened children were educated on malaria prevention and provided with a long-lasting insecticidal net. Parents or guardians provided written informed consent.

The trial protocol was approved by local and national regulatory authorities, institutional review boards, and independent ethics committees (appendix p 2). An independent data monitoring committee is overseeing the study. The trial is conducted in accordance with the principles of the Declaration of Helsinki.

### Randomisation and masking

Children were randomly assigned (1:1:1:1:1) to receive one of four different RTS,S vaccination regimens or a rabies control vaccine (M012 schedule) with an adaptive procedure using a web-based randomisation system with a minimisation procedure accounting for centre. Those in the RTS,S groups received two full doses at month 0 and month 1 and either full doses at month 2 and month 20 (group R012-20), full doses at month 2, month 14, month 26, and month 38 (group R012-14), fractional doses at month 2, month 14, month 26, and month 38 (group Fx012-14; early fourth dose), or fractional doses at month 7, month 20, and month 32 (group Fx017-20; delayed third dose; appendix p 4). After obtaining the signed consent, site staff in charge of vaccine administration confirmed trial eligibility and enrolled the child. Identification numbers were assigned sequentially to all enrolled children. Site staff entered the child's identification number in the randomisation system, which provided the study group and generated a code for the vaccine to be used for the first dose. The procedure was similar for subsequent doses.

### Procedures

The compositions of RTS,S^12^ and the rabies vaccine (manufactured by GSK, Marburg, Germany and owned by Bavarian Nordic, Hellerup, Denmark)^13^ have been previously described. The fractional doses were administered as one fifth (0·1 mL) of the full RTS,S dose after reconstitution. All vaccines were administered by intramuscular injection in the left deltoid.

We assessed *P falciparum* parasitaemia at scheduled cross-sectional visits conducted monthly up to month 20 at study clinics or children's household (active case detection). Parents or guardians were encouraged to bring the child to the study-designated health-care facility in case of illness (passive case detection) at any point in time. Blood samples for efficacy analyses were taken to prepare a blood smear for microscopy in children with fever at the time of presentation or within the previous 24 h.

The primary case definition for clinical malaria was *P falciparum* asexual parasitaemia more than 5000 parasites per μL and fever (axillary temperature ≥37·5°C)^14^ identified during passive detection. The secondary case definition was *P falciparum* asexual parasitaemia more than zero parasites per μL and fever or history of fever within 24 h of presentation (passive case detection; appendix p 8).

Incident infections were defined as first episodes of parasite densities of more than zero parasites per μL, irrespective of fever, in children with no parasitaemia at study start (active or passive case detection). Prevalent infections were defined as all episodes of parasite density of more than zero parasites per μL during cross-sectional visits (active detection; appendix p 8).

Blood slides for vaccine efficacy assessment were not read in real time. All slides (parasite detection and quantification) were read at KEMRI/United States Army Medical Research Directorate-Africa, Malaria Diagnostics Center (Kisumu, Kenya) according to the site's protocols and WHO standards were ensured. Blood films were read by two independent microscopists using a methodology previously described (counting against known blood volume), with discrepant readings resolved by a third reader.^15^, ^16^

For patient care, additional blood slides or rapid diagnostic tests were read at the respective sites in real time. We treated participants positive for malaria according to national guidelines in each country. Children were not presumptively treated for malaria at baseline.

Blood samples for immunogenicity assessments were collected as shown in appendix p 4. Anticircumsporozoite protein and anti-HBs antibody geometric mean concentrations (GMCs) and seropositivity or seroprotection rates were assessed in a subset comprising 250 children (ie, the first 25 children per country randomly assigned into each group [immunogenicity subset]). Assays are described in appendix p 9.

We analysed solicited adverse events after doses three and four in children in the reactogenicity subset (the same as the immunogenicity subset). Trained personnel collected solicited local and general adverse events on diary cards for 4 days after vaccination. All adverse events in children not in the reactogenicity subset were reported as unsolicited adverse events. We collected unsolicited adverse events for 30 days after vaccination through passive surveillance at inpatient and outpatient facilities and during home visits, from all children in the exposed set, which comprised children who received at least one vaccine dose. All solicited adverse events were graded by intensity, with grade 3 indicating severe adverse events. All solicited local adverse events were considered causally related to vaccination, and the investigators assessed the causality of all other adverse events.

Cases of severe malaria and cerebral malaria, serious adverse events, including adverse events of specific interest (meningitis and potential immune-mediated diseases), and adverse events leading to withdrawal, were reported throughout the study. Additionally, we analysed seizures occurring within 30 days after vaccination. To support diagnosis of potential immune-mediated diseases, when indicated, samples were sent to Clinical Laboratory Sciences South Africa for further testing (appendix p 10). For clinically suspected meningitis cases (appendix p 8), cerebrospinal fluid samples were tested by PCR for causal pathogens (appendix p 10) at Clinical Laboratory Sciences South Africa.

### Outcomes

The primary endpoint was the occurrence of clinical malaria meeting the primary case definition from month 2·5 up to month 14. The superiority in terms of vaccine efficacy of a M012 schedule with a fractional third RTS,S dose at month 2 (group Fx012-14) compared with the M012 schedule with full RTS,S doses (pooled R012-14 and R012-20 groups) was demonstrated if the lower limit of the 95% CI for the incremental vaccine efficacy estimate was more than zero. The first secondary endpoint was occurrence of clinical malaria meeting primary and secondary case definitions in the Fx012-14 group from month 2·5 up to month 14. Other secondary outcomes related to vaccine efficacy assessment were occurrence of clinical malaria meeting the primary and secondary case definitions in RTS,S groups at different follow-up periods up to month 20 and the prevalence and incidence of *P falciparum* infections from study start to month 20 (appendix p 3). We also present immune responses to the circumsporozoite protein and HBs antigens and safety data analysed up to month 21, when all RTS,S groups had received the fourth dose.

### Statistical analysis

The study had at least 90% power to detect significant incremental vaccine efficacy against the first or only episode of clinical malaria of the Fx012-14 regimen over the standard full-dose regimen (primary endpoint), assuming 250 evaluable children per group. Expected incidences were at least 0·5 episodes per person-year at risk in children in the control group, 0·29 episodes per person-year at risk in the pooled R012-14 plus R012-20 groups, and 0·16 episodes per person-year at risk in the Fx012-14 group (an incremental vaccine efficacy of 44·8% was assumed). The incidence of *P falciparum* infection was anticipated to be higher than that of clinical malaria, resulting in adequate power for endpoints evaluating vaccine efficacy against infections.

The primary analysis of efficacy was done in the per-protocol set, including children who received all three first vaccinations as per protocol and who contributed to efficacy surveillance starting 14 days after dose three. In groups R012-14 and Fx012-14, children not receiving dose four per protocol were censored at the last documented visit if available or at 13 months after dose one if not; these children contributed to the per-protocol analysis up to censoring. Secondary analyses were carried out in the per-protocol set, unless otherwise specified.

Vaccine efficacy estimates were calculated for an RTS,S regimen (full or fractional dose) compared with the control group, whereas incremental vaccine efficacy was estimated by comparing one RTS,S group with another. Vaccine efficacy and incremental vaccine efficacy estimates against first or only episode of incident clinical malaria or *P falciparum* infection were calculated and presented as 100 × (1 – hazard ratio) from the Cox proportional hazards model, stratified by country. Vaccine efficacy against all episodes of clinical malaria was calculated as 100 × (1 – incidence rate ratio), overall (adjusted for country as a fixed effect) and by country, and analysed by negative binomial regression allowing for interdependence between episodes within the same child.^17^

Vaccine impact was defined as the estimated number of cases of clinical malaria averted over the relevant period per 1000 children vaccinated. The estimated number of cases was calculated as the area under the 3-month incidence curve of clinical malaria for each group (sum of the differences in incidence between the RTS,S and control groups per year by 3-month periods multiplied by 1000/4). In post-hoc analyses, 95% CIs for the incidence of clinical malaria episodes by 3-month periods were computed using a generalised linear model with a Poisson distribution, the log as the link function, an offset (log time), and deviance as the scale, with the group variable as fixed covariable (factor with the four active groups and the control group being the reference group). In addition, the prevalence of *P falciparum* infections at each calendar month was calculated as the proportion of participants reporting at least one infection from the total number of participants in each group for whom parasitaemia density was available for the considered month (when considering all cross-sectional visits).

All endpoints were analysed sequentially, and any conclusion on the first secondary endpoint was conditional to reaching the primary endpoint. All other secondary endpoints should be interpreted descriptively.

All analyses were done with SAS (version 9.4) This trial is registered with ClinicalTrials.gov, NCT03276962.

### Role of the funding source

GlaxoSmithKline Biologicals was involved in study design and oversight, coordinated data collection, data analysis, data interpretation, and writing of the report. PATH's Malaria Vaccine Initiative contributed to study design and data interpretation but was not involved in data collection.

## Results

**Figure 1:**
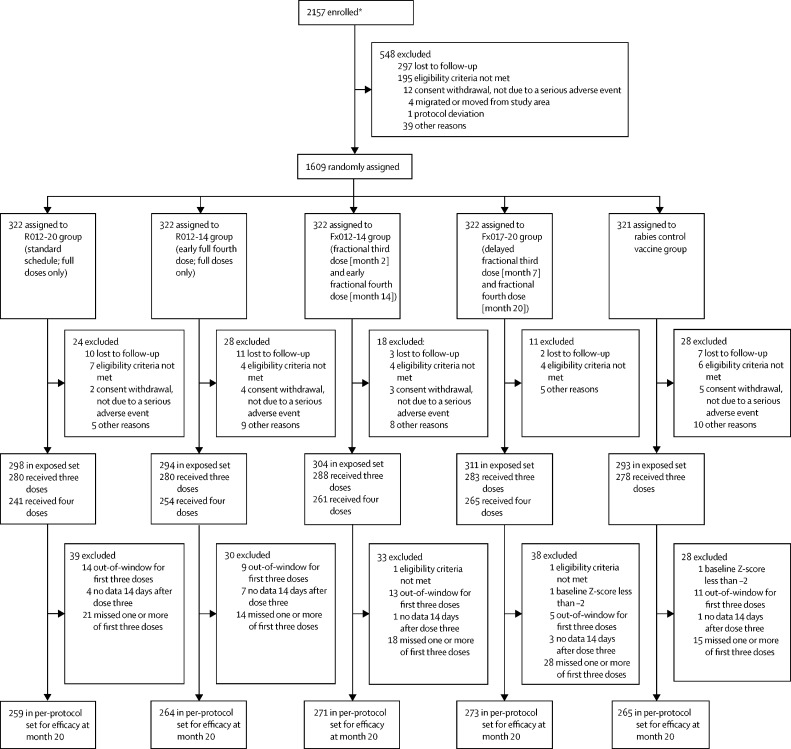
Trial profile Other reasons for exclusion included not attending first visit as scheduled (within 28 days from screening), incomplete screening procedures, one parent declining participation, Z-score less than −2, low haemoglobin concentration, moderate malnutrition, and recruitment target reached. *Signed informed consent form.

Between Sept 28, 2017, and Sept 25, 2018, 2157 children were enrolled, of whom 1609 were randomly assigned to a treatment group (322 to R012-20, 322 to R012-14, 322 to Fx012-14, 322 to Fx017-20, and 321 to the rabies vaccine control group). 1500 children received at least one study vaccine dose and the per-protocol set comprised 1332 children (figure 1). Baseline characteristics were similar across groups and countries and between the exposed set and the per-protocol set (table 1).

**Table 1:** Baseline characteristics, overall and by country

		**R012-20 group (n=322)**	**R012-14 group (n=322)**	**Fx012-14 group (n=322)**	**Fx017-20 group (n=322)**	**Control group (n=321)**
**Exposed set**						
Number in exposed set		298	294	304	311	293
	Ghana	153 (51%)	151 (51%)	148 (49%)	151 (49%)	147 (50%)
	Kenya	145 (49%)	143 (49%)	156 (51%)	160 (51%)	146 (50%)
Age at first vaccination, months		10·2 (3·9)	10·3 (3·8)	10·5 (4·0)	10·2 (3·8)	10·5 (3·9)
	Ghana	9·7 (3·9)	10·4 (4·0)	10·1 (4·0)	10·2 (4·1)	10·4 (4·0)
	Kenya	10·7 (3·7)	10·1 (3·6)	10·9 (4·0)	10·2 (3·6)	10·7 (3·8)
Male		179 (60%)	140 (48%)	132 (43%)	148 (48%)	141 (48%)
	Ghana	93 (61%)	68 (45%)	64 (43%)	76 (50%)	65 (44%)
	Kenya	86 (59%)	72 (50%)	68 (44%)	72 (45%)	76 (52%)
Female		119 (40%)	154 (52%)	172 (57%)	163 (52%)	152 (52%)
	Ghana	60 (39%)	83 (55%)	84 (57%)	75 (50%)	82 (56%)
	Kenya	59 (41%)	71 (50%)	88 (56%)	88 (55%)	70 (48%)
Length, cm		70·7 (5·3)	71·0 (5·3)	70·8 (5·2)	70·7 (5·1)	71·2 (5·3)
	Ghana	70·4 (5·6)	71·4 (5·3)	71·0 (5·6)	71·4 (5·6)	71·8 (5·4)
	Kenya	71·0 (5·0)	70·7 (5·2)	70·6 (4·7)	70·1 (4·5)	70·7 (5·1)
Bodyweight, kg		8·5 (1·3)	8·5 (1·5)	8·5 (1·4)	8·4 (1·4)	8·4 (1·3)
	Ghana	8·2 (1·4)	8·3 (1·4)	8·3 (1·4)	8·3 (1·6)	8·3 (1·4)
	Kenya	8·8 (1·3)	8·7 (1·6)	8·6 (1·3)	8·5 (1·2)	8·5 (1·3)
Baseline haemoglobin, g/dL		10·1 (1·1)	10·3 (1·1)	10·4 (1·1)	10·3 (1·1)	10·3 (1·1)
	Ghana	10·5 (1·1)	10·7 (1·0)	10·7 (1·1)	10·6 (1·0)	10·7 (1·0)
	Kenya	9·7 (1·1)	9·9 (1·1)	10·1 (1·1)	10·0 (1·0)	9·9 (1·2)
**Per-protocol set for efficacy**						
Number in per-protocol set		259	264	271	273	265
	Ghana	134 (52%)	135 (51%)	136 (50%)	141 (52%)	141 (53%)
	Kenya	125 (48%)	129 (49%)	135 (50%)	132 (48%)	124 (47%)
Age at first vaccination, months		10·3 (3·9)	10·2 (3·8)	10·3 (3·9)	10·1 (3·9)	10·5 (3·8)
	Ghana	9·7 (4·0)	10·3 (4·0)	9·8 (3·9)	10·2 (4·1)	10·3 (3·9)
	Kenya	10·9 (3·7)	10·1 (3·5)	10·8 (3·9)	10·1 (3·5)	10·8 (3·6)
Male		151 (58%)	128 (49%)	115 (42%)	131 (48%)	128 (48%)
	Ghana	77 (57%)	64 (47%)	58 (43%)	68 (48%)	60 (43%)
	Kenya	74 (59%)	64 (50%)	57 (42%)	63 (48%)	68 (55%)
Female		108 (42%)	136 (52%)	156 (58%)	142 (52%)	137 (52%)
	Ghana	57 (43%)	71 (53%)	78 (57%)	73 (52%)	81 (57%)
	Kenya	51 (41%)	65 (50%)	78 (58%)	69 (52%)	56 (45%)
Length, cm		70·8 (5·4)	70·9 (5·1)	70·6 (5·1)	70·6 (5·1)	71·4 (5·1)
	Ghana	70·4 (5·7)	71·3 (5·2)	70·7 (5·5)	71·3 (5·6)	71·8 (5·4)
	Kenya	71·2 (5·0)	70·6 (5·0)	70·5 (4·7)	69·9 (4·5)	70·9 (4·8)
Bodyweight, kg		8·4 (1·4)	8·5 (1·5)	8·4 (1·3)	8·3 (1·4)	8·4 (1·3)
	Ghana	8·2 (1·4)	8·3 (1·4)	8·2 (1·4)	8·3 (1·6)	8·3 (1·4)
	Kenya	8·7 (1·3)	8·6 (1·6)	8·6 (1·2)	8·4 (1·2)	8·5 (1·3)
Baseline haemoglobin, g/dL		10·1 (1·1)	10·3 (1·1)	10·4 (1·1)	10·3 (1·1)	10·3 (1·1)
	Ghana	10·5 (1·0)	10·7 (0·9)	10·7 (1·0)	10·6 (1·0)	10·7 (1·0)
	Kenya	9·7 (1·1)	9·9 (1·1)	10·2 (1·1)	10·0 (1·1)	9·9 (1·1)

Data are N, n (%), or mean (SD). Total numbers of participants in each group make up the enrolled set.

**Figure 2:**
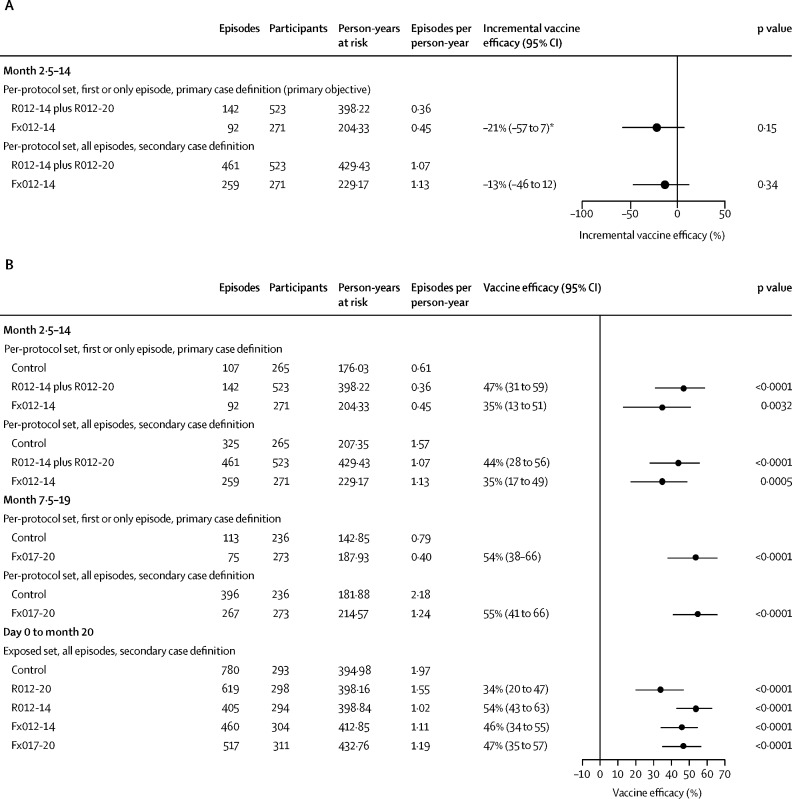
Incremental vaccine efficacy and vaccine efficacy against clinical malaria (A) Incremental vaccine efficacy in the per-protocol set. (B) Vaccine efficacy in the per-protocol set and the exposed set. *As the lower 95% CI bound was less than zero, the primary objective was not met.

During the 12 months after dose three, the incidence of the first or only episode of clinical malaria meeting the primary case definition was 0·36 per person-year in the pooled R012-14 plus R012-20 groups, 0·45 per person-year in the Fx012-14 group, and 0·61 per person-year in the control group. The incremental vaccine efficacy in the Fx012-14 group versus the pooled R012-14 plus R12-20 groups was −21% (95% CI −57 to 7; p=0·15; figure 2). Superiority of the Fx012 regimen over the full-dose R012 schedule was not shown.

Over the 12 months after dose three, vaccine efficacy against first or only episode of clinical malaria meeting the primary case definition was 47% (95% CI 31–59) in the pooled R012-14 plus R012-20 groups, 35% (13–51) in the Fx012-14 group, and 54% (38–66) in the Fx017-20 group. Vaccine efficacy estimates were similar for each group irrespective of whether they were assessed for first or only episodes or all episodes and meeting the primary or secondary case definitions (figure 2; appendix pp 11–13).

Vaccine efficacy against all episodes of clinical malaria (secondary case definition) from day 0 to month 20 after four vaccinations in the R012-14 group and the Fx012-14 group, and after three vaccinations in the R012-20 group and the Fx017-20 group is shown in figure 2. The vaccine efficacy for the Fx017-20 regimen tended to be lower than that for M012 schedules at shorter follow-up periods (7 months after dose two or three), but the difference decreased over time up to month 20 (appendix pp 11–13). An estimated 213 (7%) of the 2843 malaria cases (all episodes, secondary case definition) were co-infected with *Plasmodium malarie* (143 [5%]) or *Plasmodium ovale* (74 [3%]) by microscopy.

Over the 12 months after dose three, incremental vaccine efficacy of a third fractional dose using the secondary case definition (all episodes) was −13% (95% CI −46 to 12) in the Fx012-14 group compared with the pooled R012-14 plus R012-20 groups (figure 2). Incremental vaccine efficacy against clinical malaria was 10% (−28 to 36) in the Fx017-20 group compared with the Fx012-14 group over 12 months after dose three (appendix p 14).

Parasite density was similar between study groups (appendix p 15). Estimates of vaccine efficacy against first or only episode of incident *P falciparum* infection up to month 20 in all RTS,S groups are shown in appendix p 16. Estimates of vaccine efficacy against all episodes of prevalent *P falciparum* infections are shown in appendix p 17.

**Figure 3:**
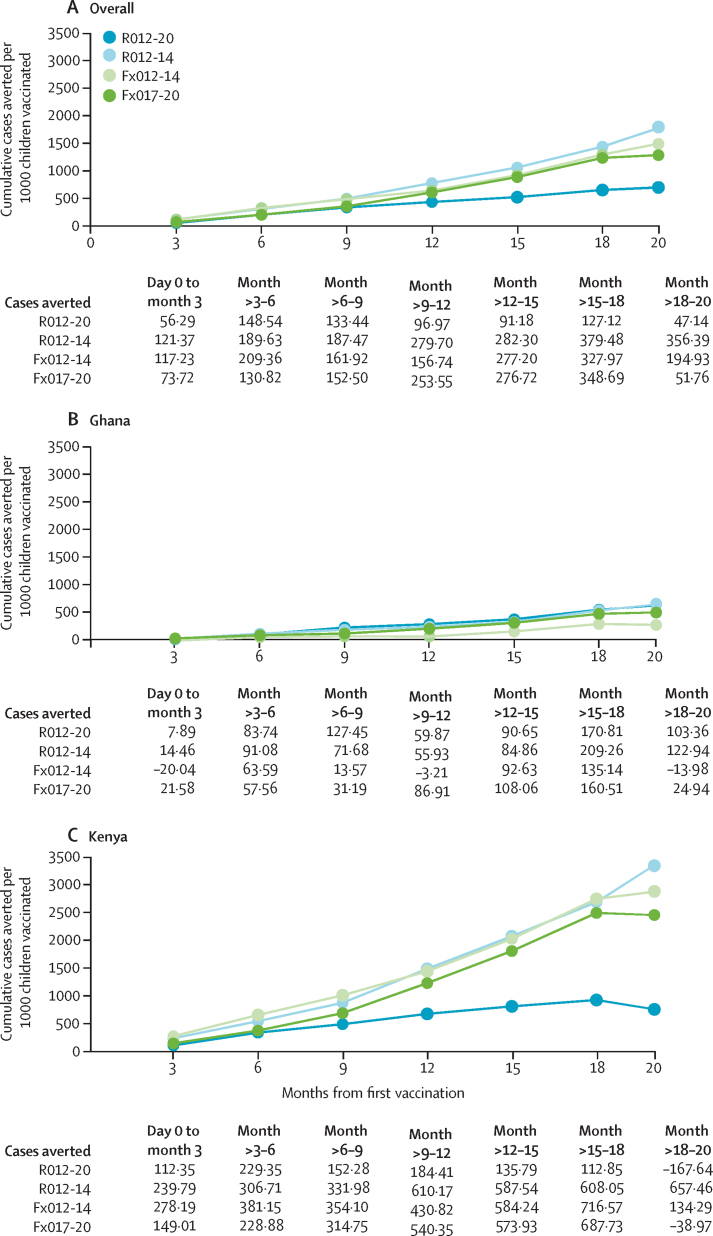
Cumulative number of averted cases of clinical malaria (secondary case definition) overall and by country, by 3-month periods per 1000 children vaccinated (exposed set) Values in the tables indicate the number of cases averted in each group over 3-month periods up to month 20.

Up to month 20, 1490 cases of clinical malaria (secondary case definition) per 1000 vaccinated children were averted in the Fx012-14 group and 1288 cases per 1000 vaccinated children were averted in the Fx017-20 group. The number of averted cases was different between the groups receiving the full-dose regimens (figure 3; appendix p 18). However, in a post-hoc analysis, the incidence of clinical malaria by 3-month periods was shown not to differ significantly (overlapping 95% CIs) between the R012-20 and R012-14 groups up to month 12 (appendix p 19).

**Figure 4:**
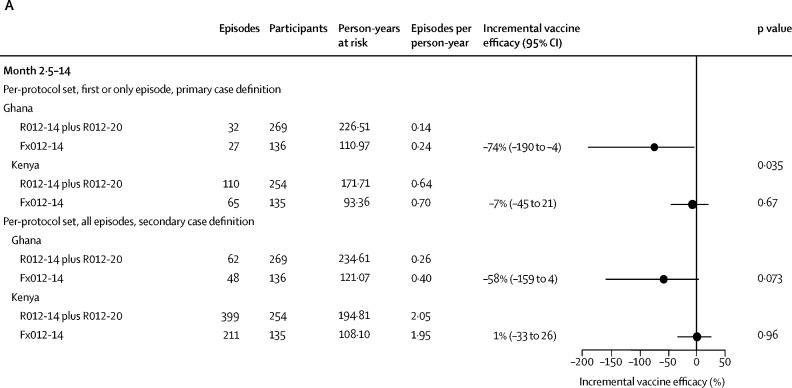

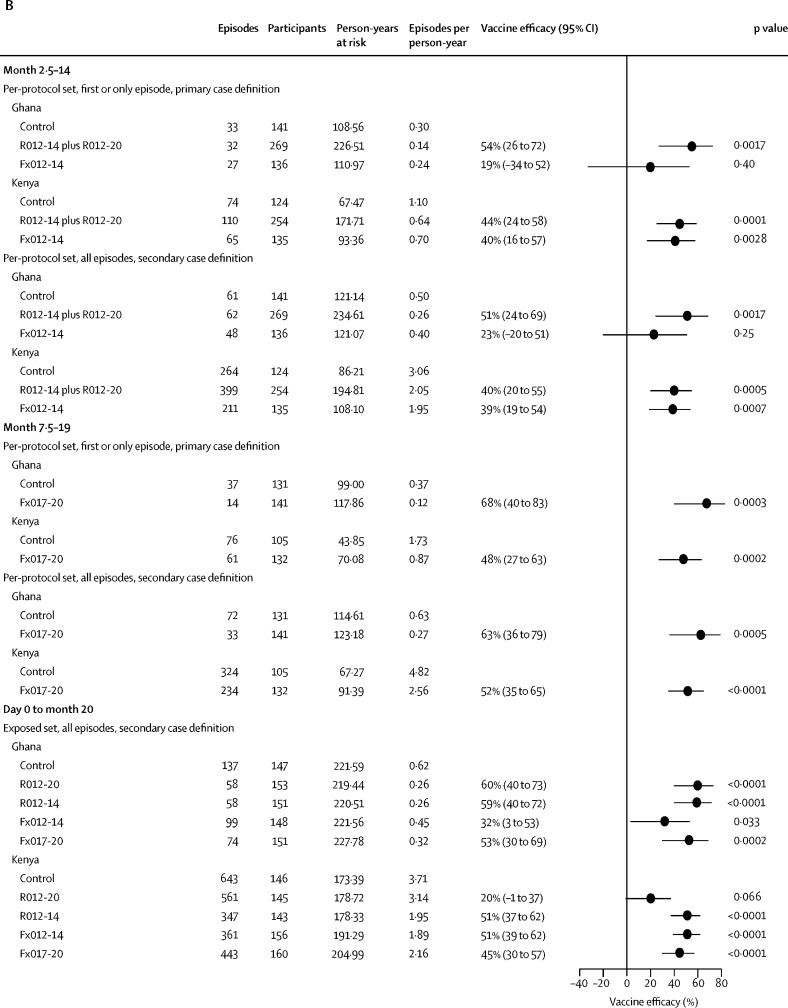
Incremental vaccine efficacy and vaccine efficacy against clinical malaria by country The trial was not powered to assess efficacy by country.

The underlying incidence of malaria differed substantially between countries, as shown by the event rates in the control groups, with Kenya consistently having more events than Ghana. The difference was more marked for all episodes (secondary case definition) than for first or only episode (primary case definition) of clinical malaria. Vaccine efficacy and incremental vaccine efficacy estimates varied between Ghana and Kenya for all endpoints (figure 4, appendix pp 11–13). Point estimates of vaccine efficacy against first or only episode of incident *P falciparum* infections tended to be higher in Ghana (where incidence was lower); the same was observed for vaccine efficacy against all episodes of prevalent infections (appendix pp 16–17).

Up to month 20, the number of clinical malaria cases averted (figure 3) in RTS,S groups was higher in Kenya where the incidence of clinical malaria cases in the control group was approximately 6 times higher compared to Ghana (figure 4).

The distribution of anti-circumsporozoite protein antibody concentrations after vaccination is shown in appendix p 5. 1 month after dose three, anti-circumsporozoite protein antibody GMCs tended to be higher in the Fx012-14 than in the Fx017-20 group but similar GMCs were observed between the groups 1 month after dose four. Overall, the fourth dose did not increase anti-circumsporozoite responses to levels observed after dose three (appendix pp 6, 20). There was no difference in anti-circumsporozoite protein antibody avidity between the fractional-dose and the full-dose regimens (appendix p 6). Anti-HBs antibody responses increased after each RTS,S administration (appendix pp 6, 20).

A summary of reported adverse events is shown in table 2. The incidence of solicited adverse events after doses three and four in the reactogenicity subset was low and similar between groups. Fever was the most frequent general adverse event, reported in both full-dose and fractional-dose groups: in five (12%) of 41 children in the R012-20 group, 11 (25%) of 44 in the R012-14 group, six (14%) of 42 in the Fx012-14 group, and two (5%) of 43 in the Fx017-20 group after dose four (table 2, appendix p 21). No clinically significant changes in haematology or biochemistry parameters were observed.

**Table 2:** Summary of adverse events

			**R012-20 group**	**R012-14 group**	**Fx012-14 group**	**Fx017-20 group**	**Control group**
Solicited adverse events over the 4-day follow-up after dose 3*†**(reactogenicity subset)**							
Solicited local adverse events							
	Erythema		0	1 (2%)	0	0	0
		Grade 3	0	0	0	0	0
	Pain		1 (2%)	1 (2%)	0	0	0
		Grade 3	0	0	0	0	0
	Swelling		0	1 (2%)	0	1 (2%)	0
		Grade 3	0	0	0	0	0
Solicited general adverse events							
	Drowsiness		2 (4%)	0	1 (2%)	2 (4%)	0
		Grade 3	0	0	0	0	0
	Irritability or fussiness		0	1 (2%)	0	3 (7%)	0
		Grade 3	0	0	0	0	0
	Loss of appetite		0	1 (2%)	0	1 (2%)	0
		Grade 3	0	0	0	0	
	Fever		12 (26%)	8 (17%)	4 (9%)	5 (11%)	1 (2%)
		Grade 3	2 (4%)	0	0	0	0
Solicited adverse events over the 4-day follow-up after dose 4*‡**(reactogenicity subset)**							
Solicited local adverse events							
	Erythema		0	1 (2%)	1 (2%)	0	..
		Grade 3	0	0	0	0	..
	Pain		1 (2%)	2 (5%)	1 (2%)	4 (9%)	..
		Grade 3	0	0	0	0	..
	Swelling		1 (2%)	1 (2%)	1 (2%)	1 (2%)	..
		Grade 3	0	0	0	0	..
Solicited general adverse events							
	Drowsiness		0	3 (7%)	2 (5%)	0	..
		Grade 3	0	0	0	0	..
	Irritability or fussiness		0	6 (14%)	1 (2%)	1 (2%)	..
		Grade 3	0	0	0	0	..
	Loss of appetite		2 (5%)	5 (11%)	3 (7%)	0	..
		Grade 3	0	0	0	0	..
	Fever		5 (12%)	11 (25%)	6 (14%)	2 (5%)	..
		Grade 3	0	1 (2%)	0	0	..
**Unsolicited adverse events, adverse events of specific interest and serious adverse events up to month 21**§**(exposed set)**							
Any unsolicited adverse event within 30 days of any vaccination			229 (77%)	231 (79%)	251 (83%)	248 (80%)	238 (81%)
	Related adverse events		16 (5%)	21 (7%)	9 (3%)	13 (4%)	6 (2%)
Adverse events of special interest							
	Meningitis		1 (<1%)	0	1 (<1%)	2 (1%)	2 (1%)
	Seizure within 30 days after vaccination		4 (1%)	0	0	4 (1%)	4 (1%)
	Potential immune-mediated disease		0	1 (<1%)	0	0	0
	Severe malaria		13 (4%)	14 (5%)	15 (5%)	20 (6%)	31 (11%)
	Cerebral malaria		0	0	0	0	1 (<1%)
Serious adverse events			48 (16%)	45 (15%)	47 (15%)	62 (20%)	71 (24%)
	Related serious adverse events		3 (1%)	0	0	2 (1%)	0
	Fatal serious adverse events		1 (<1%)	1 (<1%)	0	2 (1%)	0

Grade 3 solicited adverse events were defined as erythema or swelling >20 mm, crying when limb is moved (pain), not eating at all (loss of appetite), preventing normal everyday activities (drowsiness and irritability or fussiness), and temperature >39·0°C (fever).

* The analysis included all children within the reactogenicity subset who had safety data; n (%) indicates the number (percentage) of doses followed by at least one solicited adverse event; all adverse events in children not in the reactogenicity subset were reported as unsolicited adverse events; all solicited local adverse events were considered related to vaccination.

† Number of children with available data: R012-20 group n=46, R012-14 group n=48, Fx012-14 group n=44, Fx017-20 group n=45, control group n=49.

‡ Number of children with available data: R012-20 group n=41, R012-14 group n=44, Fx012-14 group n=42, Fx017-20 group n=43.

§ Number of children with available data: R012-20 group n=298, R012-14 group n=294, Fx012-14 group n=304, Fx017-20 group n=311, control group n=293; n (%) indicates the number (percentage) of children with at least one adverse event.

Unsolicited adverse events occurring within 30 days from any vaccination up to month 21 are shown in table 2 and appendix p 22, with upper respiratory tract infection (in 161 [54%] of 298 children in the R012-20 group, 175 [60%] of 294 in the R012-14 group, 184 [61%] of 304 in the Fx012-14 group, 181 [58%] of 311 in the Fx017-20 group, and 164 [56%] of 293 in the control group) and gastroenteritis (in 57 [19%] children in the R012-20 group, 81 [28%] in the R012-14 group, 72 [24%] in the Fx012-14 group, 67 [22%] in the Fx017-20 group, and 72 [25%] in the control group) being the most frequently reported.

Serious adverse events up to month 21 were reported in 48 (16%) children in the R012-20 group, 45 (15%) in the R012-14 group, 47 (15%) in the Fx012-14 group, 62 (20%) in the Fx017-20 group, and 71 (24%) in the control group (table 2, appendix p 22). Four deaths, unrelated to vaccination, were reported, caused by drowning in one boy (R012-20 group), gastroenteritis in two girls (R012-14 and Fx017-20 groups), and a wall falling on a boy (Fx017-20 group).

Malaria cases up to month 21 were reported in 89 (30%) children in the R012-20 group, 86 (29%) in the R012-14 group, 84 (28%) in the Fx012-14 group, 100 (32%) in the Fx017-20 group, and 101 (35%) in the control group (appendix p 22). Severe malaria was reported in 13 (4%) of 298 children in the R012-20 group, 14 (5%) of 294 in the R012-14 group, 15 (5%) of 304 in the Fx012-14 group, 20 (6%) of 311 in the Fx017-20 group, and 31 (11%) of 293 in the control group. Cerebral malaria was reported in one (<1%) child in the control group.

Cause-confirmed viral meningitis was detected in six children: one (<1%) each in the R012-20 and Fx012-14 groups and two (1%) each in the control and Fx017-20 groups. At least one seizure episode within 30 days after any vaccination occurred in 12 children (four [1%] in each of the R012-20, Fx017-20, and control groups). Convulsive seizures levels 1–2 (according to the Brighton Collaboration Working Group case definition for generalised convulsive seizures)^18^ within 7 days of any vaccination were reported for four (1%) children in the R012-20 group, four (1%) in the Fx017-20 group, and two (<1%) in the control group. A potential immune-mediated disease (blister; verbatim term bullous skin disease) occurring 245 days after dose three (R012-14 group) was not considered related to vaccination.

**Figure 5:**
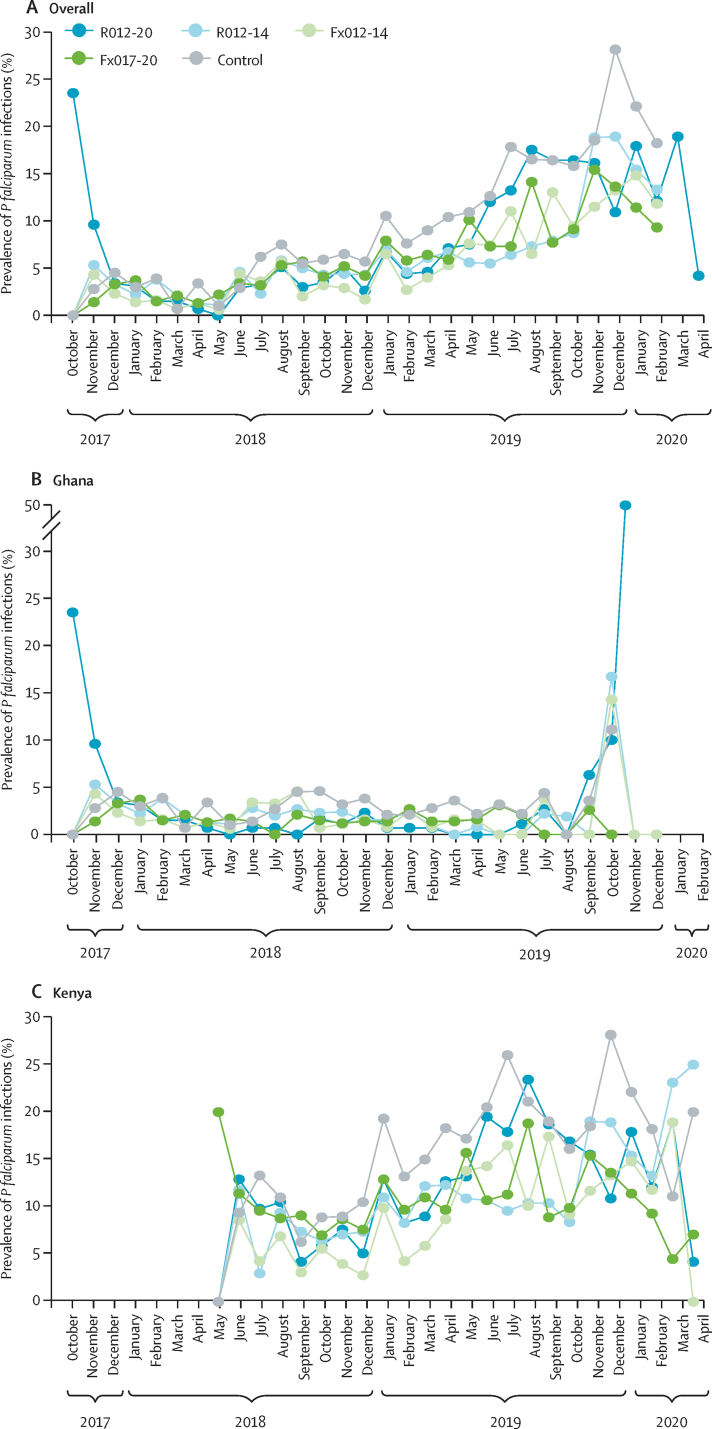
Prevalence of *Plasmodium falciparum* infections by calendar month, overall and by country (exposed set) For improved clarity, the months of May and June, 2020, are not included in these charts, as the prevalence of *P falciparum* infections was 0.

A post-hoc analysis showed that the prevalence of *P falciparum* infections varied greatly by calendar month from enrolment to study month 21, with no clear seasonal pattern observed (figure 5). Due to the difference in the start of enrolment, the overall prevalence reflects mainly data from Ghana over the first study months and from Kenya in the last study months. The greater variability observed in the earliest and latest calendar months was due to a lower number of children at risk or the total number of children included in the analyses. The prevalence of *P falciparum* infections at cross-sectional visits is shown in appendix p 7.

## Discussion

To our knowledge, this is the first study to assess whether regimens including fractional doses increase the protective efficacy and impact of RTS,S against malaria in countries with moderate-to-high malaria endemicity. Our findings show the use of a fractional instead of a full third RTS,S dose does not provide superior vaccine efficacy when children aged 5–17 months are vaccinated according to the standard M012 schedule. Nevertheless, during the 20-month period of this interim analysis, vaccine efficacy for each RTS,S group versus control was consistent with efficacy results from previous clinical trials.^2^, ^3^, ^19^

Our findings differ from previous observations from a CHMI trial in malaria-naive adults, which suggested improved efficacy of the vaccine with fractional dosing.^7^ However, these CHMI studies evaluated vaccine efficacy against *P falciparum* infection following homologous challenge with high-density sporozoite infected laboratory-raised mosquitos.^6^, ^7^, ^20^ This vaccine efficacy estimate cannot fully translate into efficacy against clinical disease in field settings where exposure might be heterologous and is the result of multiple bites from wild mosquitoes with potentially lower sporozoite density infections. In a trial in 6–10-week-old infants from Ghana, Tanzania, and Gabon, when delaying the administration of the third full dose from month 2 to month 7, no efficacy was observed before the third dose, and no improvement of vaccine efficacy against clinical malaria was noted over a period of 19 months of follow-up from first dose.^21^

Moreover, it was previously hypothesised, based on germinal-centre and B-cell biology,^22^ that in the context of challenge studies in malaria-naive adults, the use of a fractional instead of a full RTS,S dose might have enhanced competitive antigen binding in germinal centres. This would have led to preferential survival and expansion of circumsporozoite-specific B cells with the highest antigen affinity and to the higher antibody avidity observed in CHMI trials. However, such improved avidity was not observed in the current study, possibly because in children constantly exposed to malaria parasites through natural infection, B-cell affinity maturation might be altered,^23^ resulting in the loss of the advantage of the fractional dose regimen seen in CHMI studies.

Overall, over the full 20-month follow-up period, substantial protection was observed in all RTS,S groups across the various endpoints assessed. The lowest vaccine efficacy was seen in the R012-20 group (in which the fourth dose given at month 20 did not contribute to the assessed efficacy as it was given at the end of the follow-up period). However, this was not the case over the initial 12 months of follow-up after dose three, when vaccine efficacy point estimates were very similar across groups. Interestingly, vaccine efficacy in the delayed fractional-dose group (Fx017-20) was similar to that in the other groups despite the 5-month delay in the administration of the third dose and the fact that the fourth dose (given at month 20) also did not contribute to the assessed efficacy. This might indicate either that the fractional RTS,S dose can be delayed by 5 months without losing protection against malaria, or that the initial lower protection in the Fx017-20 group after dose two is compensated by a higher efficacy of the delayed third fractional dose.

We observed a difference in the background incidence of clinical malaria between Ghana and Kenya, similar to previous reports for malaria incidence in areas close to the study sites.^24^ A trend for lower vaccine efficacy of RTS,S with higher malaria incidence was previously described, but a statistical proof of an interaction between vaccine efficacy and transmission intensity could not be established.^3^, ^19^ In our study, we also observed higher point estimates of efficacy with lower background malaria incidence in Ghana (except in the Fx012-14 group) over the 20-month follow-up period. However, our study was not designed to assess differences between countries or country-specific data, thus observed differences could also be due to chance.

Over the initial 12 months of follow-up after dose three, there was a discrepancy between groups receiving the same full-dose regimen (R012-20 and R012-14) when assessing vaccine efficacy against all episodes of clinical malaria. This discrepancy is driven by results from Kenya. However, a post-hoc analysis showed that, during this period, the incidence of clinical malaria episodes did not differ significantly between the R012-20 and R012-14 groups, although point estimates were lower in the R012-14 group than the R012-20 group. Therefore, we concluded that this was most probably a chance finding, which could nevertheless have contributed to the overall efficacy over 20 months of follow-up. Therefore, any conclusion on the potential benefit of a fourth dose given 12 months after dose three compared with 18 months after dose three should be drawn with caution this early in the study.

Anti-circumsporozoite protein antibody responses to RTS,S were similar between groups. Consistent with previous observations, the fourth dose did not boost anti-circumsporozoite protein antibodies to higher concentrations than those achieved after dose three.^3^, ^21^ This is in contrast to the anti-HBs responses that show an incremental response after each dose, with booster responses largely exceeding the antibody concentration after dose three.

In this study, we did not observe a difference in antibody avidity between the fractional-dose and full-dose regimens. In a previous CHMI study, the delayed fractional dose was shown to increase circumsporozoite protein-specific antibody avidity, which was hypothesised to be a potential contributor to the improved vaccine efficacy of an Fx017 regimen over an R012 regimen.^7^, ^25^ However, in a more recent CHMI challenge trial, anti-circumsporozoite protein-specific antibody avidity was not different between protected and unprotected individuals.^6^ The avidity of anti-NANP IgG responses elicited by RTS,S administered according to full-dose R012 and R017 regimens was not shown to be associated with protection from clinical malaria in children enrolled in two phase 2 field trials.^26^, ^27^ By contrast, anti-circumsporozoite protein IgG concentrations and avidity, which varied with age, site, and prevaccination concentrations, were shown to contribute to protection against clinical malaria in the RTS,S phase 3 efficacy trial.^28^

All regimens were well tolerated, supporting the acceptable safety profile of RTS,S. The incidence of solicited adverse events was similar to that observed in the phase 3 trial assessing the full-dose M012 regimen in African children aged 5–17 months.^2^ Meningitis and cerebral malaria were previously highlighted as safety signals in the RTS,S phase 3 trial.^3^, ^29^ We did not observe any indication of increased risk for suspected or cause-confirmed meningitis in the RTS,S groups compared with the control group, and the only case of cerebral malaria was reported in the control group. This is in line with recent findings from the ongoing pilot implementation of RTS,S.^30^

Our study has several limitations. Because the primary objective was not demonstrated, any further group comparisons should be interpreted with caution. Comparison between the delayed fractional regimen and groups receiving a M012 schedule might be hindered by seasonal variations in malaria incidence and transmission; in addition, the study did not include a R017-20 full-dose group to allow a non-biased comparison with the fractional Fx017-20 regimen. The study was only powered for overall results and not by site; this, together with the heterogeneity observed for vaccine efficacy estimates, known difference in malaria transmission even within the sites, and the multiplicity of objectives, limit the interpretation of results by country. Due to the open-label design, some bias in safety assessments is possible, although the study was designed to minimise it (ie, vaccinations and safety assessments were done by different individuals).

In conclusion, the Fx012-14 regimen did not show superior vaccine efficacy against clinical malaria after the three first doses when compared with a standard full-dose M012 schedule, and improved vaccine efficacy was also not observed for the delayed Fx017-20 regimen. However, all fractional-dose and full-dose RTS,S regimens provided substantial and similar protection against malaria.

Our results suggest that the use of a delayed fractional-dose regimen does not affect protective efficacy over 20 months of follow-up compared with a standard regimen, and that the timing of the third and fourth RTS,S vaccinations might be flexible. Although not confirmatory, if borne out over the 50-month follow-up, these findings might portend substantial public health benefit, as reduced vaccine volume requirements and flexibility in dose timing would allow increased access to the vaccine and decreased malaria-specific morbidity and mortality. Continued follow-up to month 50 will provide further insight into the efficacy of different RTS,S regimens, including the effect of multiple annual booster doses.

## Data sharing

Anonymised individual participant data and study documents will be available at study end, when they can be requested for further research from www.clinicalstudydatarequest.com (study ID 204889).

## Declaration of interests

The findings and conclusions in this report are those of the authors and do not necessarily represent the official position of the US Centers for Disease Control and Prevention. OO-A, LS, ML, DM, ABo, FR, and EJ are employees of the GSK group of companies. OO-A, LS, DM, FR, and EJ have restricted shares in the GSK group of companies. All other authors declare no competing interests.
