# Correction to *Lancet Infect Dis* 2022; 22: 1329–42

**DOI:** 10.1016/S1473-3099(22)00610-7

**Published:** 2022-11

*Samuels AM, Ansong D, Kariuki SK, et al. Efficacy of RTS,S/AS01_E_ malaria vaccine administered according to different full, fractional, and delayed third or early fourth dose regimens in children aged 5–17 months in Ghana and Kenya: an open-label, phase 2b, randomised controlled trial.* Lancet Infect Dis *2022; **22:** 1329–42*—This Article had incorrect copyright information. This correction has been made to the online version as of Sept 9, 2022.

