# Supplementary material for: Efficacy of RTS,S/AS01E malaria vaccine administered according to different full, fractional, and delayed third or early fourth dose regimens in children aged 5–17 months in Ghana and Kenya: an open-label, phase 2b, randomised controlled trial
Source: Lancet Infect Dis. 2022 Sep;22(9):1329–42. doi: 10.1016/S1473-3099(22)00273-0 (PMC9420828; doi:10.1016/S1473-3099(22)00273-0)
Supplement: Supplementary appendix [file mmc1.pdf]

THE LANCET
Infectious Diseases

Supplementary appendix

This appendix formed part of the original submission and has been peer reviewed. We post it as supplied by the authors.

Supplement to: Samuels AM, Ansong D, Kariuki SK, et al. Efficacy of RTS,S/$AS01_E$ malaria vaccine administered according to different full, fractional, and delayed third or early fourth dose regimens in children aged 5–17 months in Ghana and Kenya: an open-label, phase 2b, randomised controlled trial. *Lancet Infect Dis* 2022; published online June 23. https://doi.org/10.1016/S1473-3099(22)00273-0.

Supplementary appendix

Supplement to Samuels AM, Ansong D, Kariuki SK. et al. RTS,S/AS01$_E$ malaria vaccine efficacy when administered according to different full- and fractional-dose and delayed third or early fourth dose regimens in children aged 5–17 months: First results from a phase 2b open-label, randomised, controlled trial in Ghana and Kenya

Text S1. Inclusion/exclusion criteria for enrolment in the study and site-specific procedures for enrolment, screening and ethics approval

I. Inclusion/exclusion criteria

All children had to satisfy ALL the following criteria at study entry:

- Children's parent(s)/legally acceptable representative(s) [LAR(s)] who would comply with the requirements of the protocol, in the opinion of the investigator
- Signed or thumb-printed and witnessed informed consent was obtained from the parent(s)/LAR(s) of the child. Where parent(s)/LAR(s) were illiterate, the consent form was countersigned by an independent witness.
- Male or female between, and including, five and 17 months of age at the time of the first vaccination.
- Healthy children (as established by medical history and clinical examination).
- Previously received three documented doses of diphtheria, tetanus, pertussis, and hepatitis B vaccine, and at least three doses of oral polio vaccine.

The following criteria were checked at the time of study entry. If ANY exclusion criterion applied, the child was not included in the study:

- Child in care: A child who has been placed under the control or protection of an agency, organization, institution, or entity by the courts, the government or a government body, acting in accordance with powers conferred on them by law or regulation. The definition of a child in care can include a child cared for by foster parents or living in a care home or institution, provided that the arrangement falls within the definition above. The definition of a child in care does not include a child who is adopted or has an appointed legal guardian.
- Off-label use of a drug or vaccine (defined as use not approved for the indication by the Food and Drug Administration or European Union member state or World Health Organization [with respect to prequalification]) other than the study vaccines, within the previous 30 days before first vaccination, or planned use during the study.
- Any medical condition making intramuscular injection unsafe.
- Chronic administration (defined as >14 days) of immunosuppressants or other immune-modifying drugs within six months prior to first vaccination. For prednisone, this meant 0.5 mg/kg/day. Inhaled and topical steroids were allowed.
- Planned administration/administration of a vaccine not foreseen by the study protocol within seven days before and after each vaccine dose.
- Concurrent participation in another clinical study, at any time during the study period, in which the child will be exposed to an investigational or a non-investigational vaccine/product (pharmaceutical product or device).
- Any confirmed or suspected immunosuppressive or immunodeficient condition, based on medical history and physical examination (no laboratory testing required).
- Family history of congenital or hereditary immunodeficiency.
- History of any reaction or hypersensitivity likely to be exacerbated by any component of the vaccines.
- History of anaphylaxis post-vaccination.
- History or documentation of a serious adverse reaction to rabies vaccination; contraindication to rabies vaccination.
- Major congenital defects.
- Serious chronic illness.
- History of a neurological disorder or atypical febrile seizure.
- Acute disease and/or fever at the time of enrolment.
  - Fever is defined as temperature ≥37.5°C for oral, axillary or tympanic route, or ≥38.0°C for rectal route.
  - Children with a minor illness (such as mild diarrhoea, mild upper respiratory infection) without fever may be enrolled at the discretion of the investigator.
- Administration of immunoglobulins and/or any blood products within three months prior to first vaccination or planned administration during the study period.
- Moderate or severe malnutrition at screening.
- Haemoglobin concentration <8 g/dL at screening.
- Same sex twins (to avoid misidentification).
- Maternal death.
- Prior receipt of an investigational malaria vaccine.

II. Study enrolment, screening and ethics approval

Prior to study start, an information campaign was held with key community leaders. In Ghana, these were administrative and senior leaders of the community, as well as health personnel from the different health facilities in the study area. In Kenya, information about the study was given as part of the Health and Demographic Surveillance System (HDSS) activities and during community meetings such as chiefs' barazas, established local Community Advisory Board and opinion leaders' meetings, women and men groups, church groups. The study brochure was posted at the participating health facilities and at strategic locations such as community halls and facility noticeboards.

In Ghana, potentially eligible children were identified at post-natal clinics. Mothers were given study information during post-natal clinic visit and were given ample time to discuss the study with their spouses before consenting. Following this, the mothers who had expressed an interest in the study were contacted and individual informed consent was sought. In Kenya, children within the eligible age category were identified through the HDSS. Prior to enrolment, a study brochure was distributed by field workers and other study staff to parents/LARs of potential participants in the community. At the participating health facilities, the study brochure was read to parent(s)/LAR(s) of eligible children and individual consent sought.

At the screening visit, a study identification card with each child picture with his or her parent/LAR was prepared and given to the child's parent(s)/LAR(s). This card contained the name of the study, the contact person at the study site, and the child's identification number. In Kenya, the study card also contained the child's HDSS numbers. The study identification card was checked at each clinic visit and used to identify study children at unscheduled visits as well.

Vaccinations took place at the maternal and child health clinic in Agogo, located close to the Presbyterian Hospital in Ghana and at the Siaya County Referral Hospital and at the study health facilities in Kenya.

Institutional review boards/independent ethics committees approved the trial protocol: the Committee for Human Research Publication and Ethics at the University Education at Kwame Nkrumah University of Science and Technology, the Ghana Health Services Ethics Review Committee, the Ghana Food and Drugs Authority, the Kenya Medical Research Institute Scientific and Ethics Review Unit (SSC #3564), the Western Institutional Review Board, the United States Centers for Disease Control and Prevention Institutional Review Board (Protocol #7058), and the Kenya Pharmacy and Poisons Board.

Text S2. Summary of study objectives and endpoints related to vaccine efficacy, assessed up to study month 20

Objective	***Endpoint***
**Primary objective**	
To demonstrate the superiority of a 3–dose schedule of RTS,S/AS01$_E$ with a fractional third dose at M2 compared to a standard schedule of RTS,S/AS01$_E$ with three full doses in terms of VE against clinical malaria (primary case definition) over 12 months post–dose 3.*	The occurrence of clinical malaria meeting the primary case definition from M2.5 up to M14.
**Secondary objectives**	
Clinical malaria	
1. To assess the IVE against clinical malaria of a schedule with a fractional third dose at M2 versus a schedule with 3 full doses (primary and secondary case definitions) over 12 months post–dose 3.<br>2. To assess the IVE against clinical malaria, over 7 and 12 months post–dose 3, of a schedule with a fractional third dose at M7 versus a schedule with a fractional third dose at M2.<br>3. To assess the IVE against clinical malaria, over 7 and 12 months post–dose 3, of a schedule with a fractional third dose at M7 versus a schedule with 3 full doses.<br><br>4. To assess the VE and impact of each RTS,S/AS01$_E$ schedule by measuring the efficacy against clinical malaria at M14, M20.	The occurrence of clinical malaria meeting the primary and secondary case definitions
Prevalent P. falciparum infections	
7. To assess the prevalence of *P. falciparum* infections of each RTS,S/AS01$_E$ schedule at cross–sectional visits (monthly from M0 to M20).	The prevalence of *P. falciparum* infections (all episodes) defined by positive blood slide at each cross-sectional survey from D0 to M20.
Incident P. falciparum infections	
8. To assess the VE against incident *P. falciparum* infections defined by positive blood slide.	The occurrence of incident *P. falciparum* infections (first or only episode) from D0 to M20.

M, month; VE, vaccine efficacy; IVE, incremental vaccine efficacy.

Note: * IVE was estimated by comparing one RTS,S/AS01$_E$ group to another. The term VE refers to vaccine efficacy estimate for a RTS,S/AS01$_E$ regimen (full- or fractional-dose) as compared with the control group. In order to control alpha not only for the primary objective but also for the secondary objective of IVE over the 12-month period post-dose 3 of a schedule with a fractional dose at month 7 versus 3 full doses, a sequential approach was implemented (hierarchical testing). The endpoints were analyzed sequentially and any conclusion on the secondary endpoint is conditional to reaching the primary endpoint. All other secondary endpoints should be interpreted descriptively.

Figure S1. Study design overview

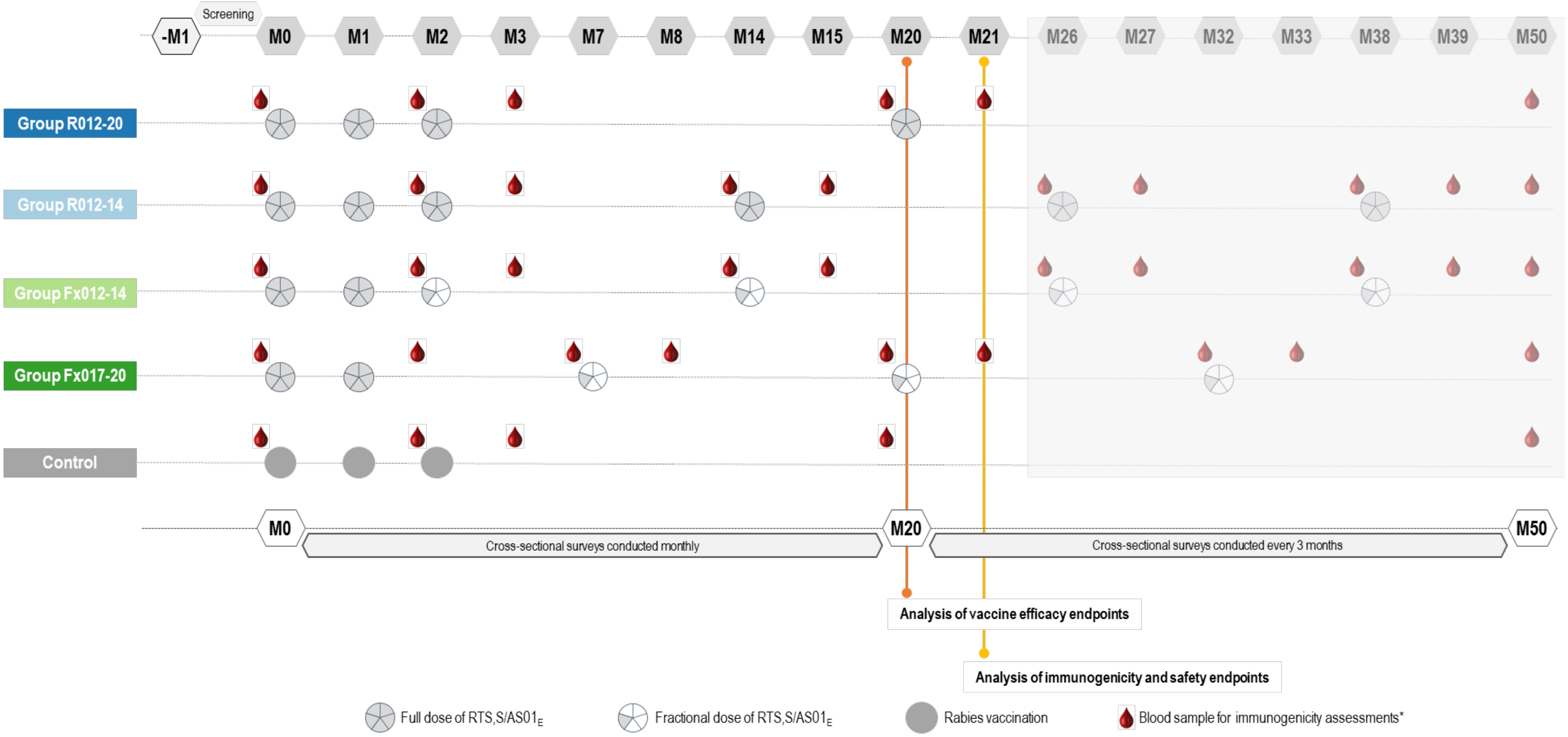

M, month.

Note: * Additional blood samples were collected in the reactogenicity/immunogenicity subset (first 50 children [25 per country] randomised into each group), for the evaluation of biochemistry and haematology parameters (pre-dose 3, seven days post-dose 3 and 30 days post-dose 3).

Full circles indicate the administration of a full dose; partially full circles symbolise a fractional dose (1/5th of the full dose). Orange and light orange lines indicate the timepoints at which analyses reported in the current manuscript were carried out. The greyed-out area indicates the study period for which results are not yet available.

Figure S2. Reverse cumulative distributions of anti-CS antibody concentrations one month post-dose 3 (A) and post-dose 4 (B) (immunogenicity subset, per-protocol set for immunogenicity)

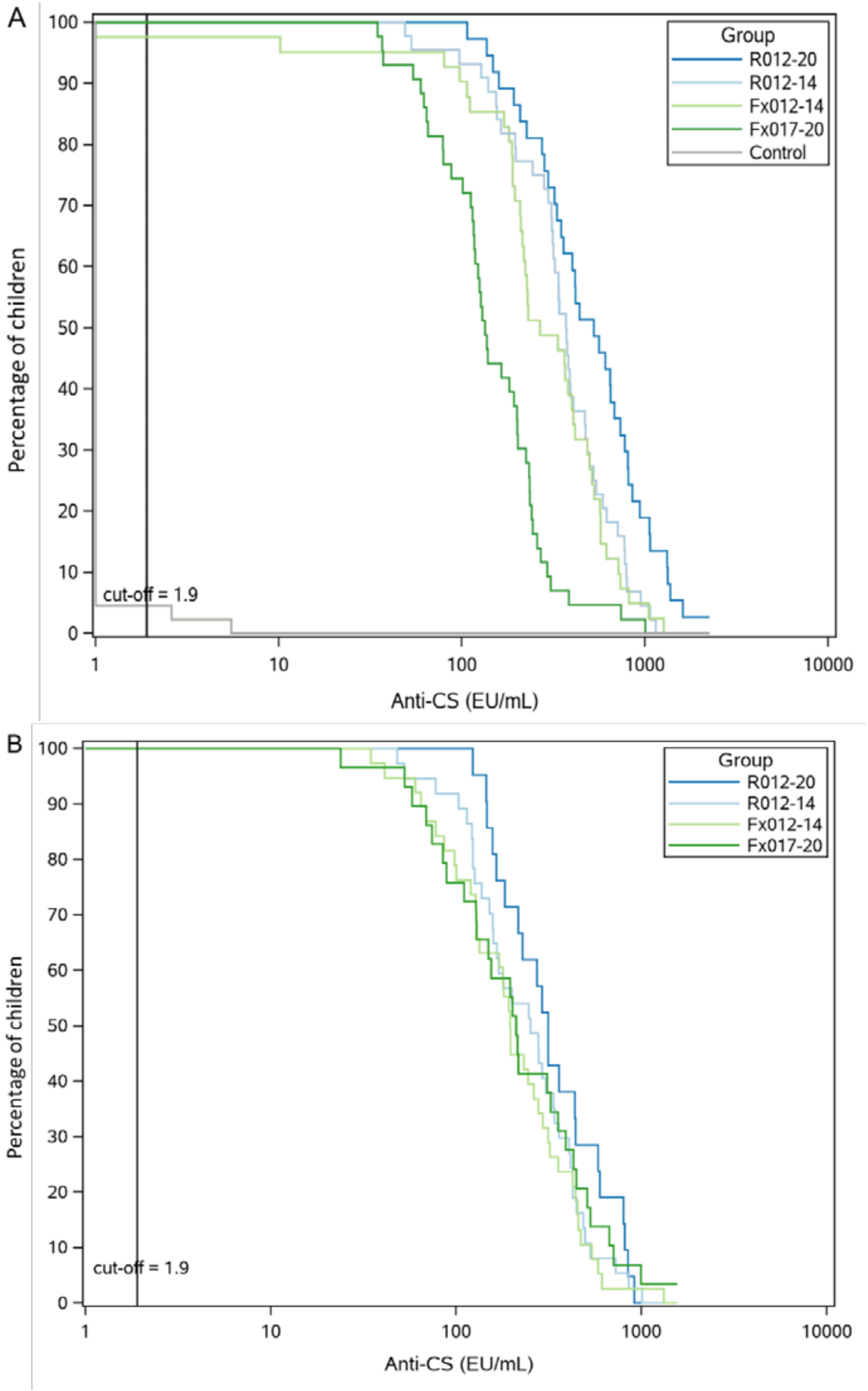

CS, circumsporozoite protein; EU, enzyme-linked immunosorbent assay units.

Figure S3. Immune responses to vaccination: anti-CS antibody GMCs (A, A’), anti-HBs antibody GMCs (B, B’), and kinetics of anti-CS antibody avidity index (C, C’), by timepoint and by month from first dose (immunogenicity subset, per-protocol set for immunogenicity)

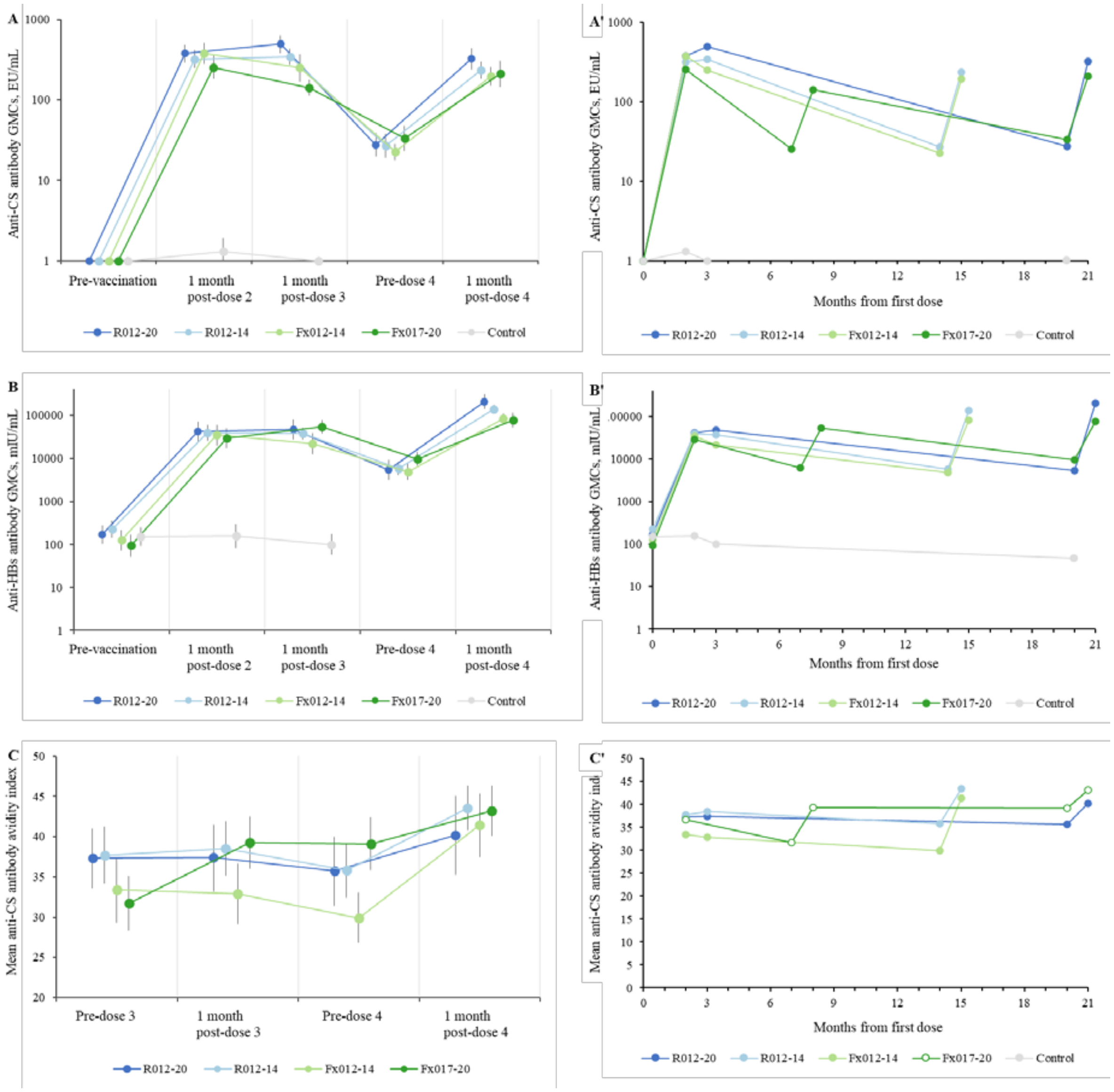

CS, circumsporozoite protein; GMC, geometric mean concentration; EU, enzyme-linked immunosorbent assay units; HBs, anti-hepatitis B surface; IU, international units.

Note: Error bars represent 95% confidence intervals.

Figure S4. Prevalence of *P. falciparum* infections at each cross-sectional visit

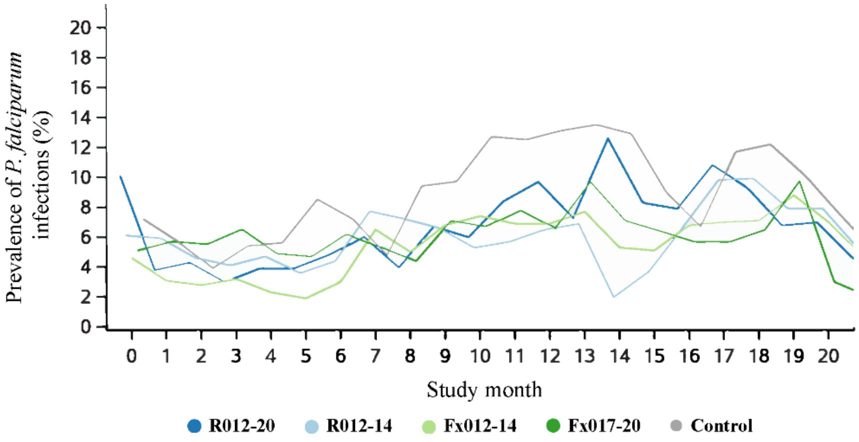

Figure S5. Plain language summary

What is the context?

- The RTS,S/AS01$_E$ vaccine acts against *Plasmodium falciparum*, the most common malaria parasite in Africa.
- RTS,S/AS01$_E$ is currently the only vaccine recommended by the World Health Organization for use in children living in areas with moderate and high risk of malaria.
- RTS,S/AS01$_E$ was moderately efficacious against clinical malaria (around 55% over 12 months of follow-up) when given as per the standard regimen (three doses one month apart) to children aged 5–17 months.
- Several controlled malaria infection trials in adults have shown that protection against *P. falciparum* infection can be increased by using other RTS,S/AS01$_E$ regimens where one or more doses are reduced (fractional-dose regimens) and administered at a different time compared to the standard regimen.

What is new?

- We are conducting a field efficacy trial in children from malaria-endemic countries, Ghana and Kenya, to evaluate the impact of fractional-dose RTS,S/AS01$_E$ regimens.
- We compare regimens with the third and fourth RTS,S/AS01$_E$ doses reduced and administered earlier, delayed or at the same time as for full-dose regimens and with the administration of a control rabies vaccine.
- During the first year after the third RTS,S/AS01E dose, the fractional-dose regimen did not show superior vaccine efficacy compared with the standard full-dose regimen.
- Up to study month 20, all fractional- and full-dose RTS,S/AS01$_E$ regimens prevented a significant number of clinical malaria cases compared with the control vaccine, with vaccine efficacies varying from 34% to 54%.
- Up to study month 21, all regimens were immunogenic and well tolerated.

What is the impact?

- All investigated regimens provided substantial protection against clinical malaria. These initial findings suggest that vaccination with RTS,S/AS01$_E$ may allow flexibility in terms of dose amount administered and schedule after the first two doses, thus potentially increasing vaccine availability.

Table S1. Case definitions used in the study

**Case**	**Definition**
Primary case definition	*P. falciparum* asexual parasitemia > 5000 parasites/μL AND presence of fever (axillary temperature ≥37.5°C) at the time of presentation AND occurring in a child who is brought for treatment to a healthcare facility.
Secondary case definition	*P. falciparum* asexual parasitaemia > 0 parasites/μL AND presence of fever (axillary temperature ≥37.5°C) at the time of presentation or history of fever within 24 hours of presentation AND occurring in a child who is brought for treatment to a healthcare facility.
Incident *P. falciparum* infection*	A documented *P. falciparum* asexual parasite density >0 parasites/μL detected by blood slide reading at a cross-sectional survey or as captured by the secondary case definition of clinical malaria (active detection of infection and passive case detection of infection).
Prevalent *P. falciparum* infection	A documented *P. falciparum* asexual parasite density >0 detected by blood slide reading at a cross-sectional visit (active detection of infection).
Severe *P. falciparum* malaria**	*P. falciparum* parasitaemia > 0 detected by microscopy and/or rapid diagnostic test<br>AND one or more of the following, occurring in the absence of an identified alternative cause:<br>• Impaired consciousness: a Glasgow coma score <11 in children two years of age or older or a Blantyre coma score <3 in children less than two years of age;<br>• Prostration: generalized weakness so that the person is unable to sit, stand or walk without assistance;<br>• Multiple convulsions: more than two episodes within 24 hours;<br>• Acidosis: a base deficit of >8 mEq/L or, if not available, a plasma bicarbonate level of <15 mmol/L or venous plasma lactate ≥5 mmol/L. Severe acidosis manifests clinically as respiratory distress (rapid, deep, laboured breathing).<br>• Hypoglycaemia: blood or plasma glucose <2.2 mmol/L (<40 mg/dL);<br>• Severe malarial anaemia: haemoglobin concentration ≤ 5 g/dL or a haematocrit of ≤ 15% in children <12 years of age with a parasite count >10000/μL;<br>• Renal impairment: plasma or serum creatinine >265 μmol/L (3 mg/dL) or blood urea >20 mmol/L;<br>• Jaundice: plasma or serum bilirubin >50 μmol/L (3 mg/dL) with a parasite count >100000/μL;<br>• Pulmonary oedema: radiologically confirmed or oxygen saturation <92% on room air with a respiratory rate >30/min, often with chest indrawing and crepitations on auscultation;<br>• Significant bleeding: including recurrent or prolonged bleeding from the nose, gums or venipuncture sites; hematemesis or melaena;<br>• Shock: compensated shock is defined as capillary refill ≥3 s or temperature gradient on leg (mid to proximal limb), but no hypotension. Decompensated shock is defined as systolic blood pressure <70 mm Hg in children, with evidence of impaired perfusion (cool peripheries or prolonged capillary refill);<br>• Hyperparasitaemia: *P. falciparum* parasitaemia >10% (i.e. percentage of infected red blood cells >10%; corresponding to >500000/μL).
Cerebral *P. falciparum* malaria*	Severe *P. falciparum* malaria with coma (Blantyre coma score < 3);<br>AND If malaria with seizure: coma persisting for > 30 min after the seizure.<br>Other treatable causes of coma should be excluded before diagnosing cerebral malaria (e.g. hypoglycaemia, bacterial meningitis).
Clinically suspected meningitis	A child with sudden onset of fever and one of the following signs: neck stiffness, altered consciousness not due to an alternative more probable cause, or other meningeal sign such as bulging fontanelle in children less than one year of age.
Confirmed meningitis	A child with clinically suspected meningitis with confirmatory evidence of meningitis from cerebrospinal fluid examination.
Aetiology-confirmed meningitis	A child with confirmed meningitis and evidence from cerebrospinal fluid examination of a specific causative agent

Note: *The analysis was performed only in children with no parasitaemia (*P. falciparum* asexual parasitaemia =0 parasites/μL) at the start of the follow-up period (D0).
** Adapted from World Health Organization. Guidelines for the treatment of malaria – 3rd edition. 2015. http://apps.who.int/iris/handle/10665/162441.

Table S2. Assessments of humoral immunity and *P. falciparum* parasitaemia

Component	Method	Kit/ Manufacturer	Technical cut–off	Laboratory
P. falciparum circumsporozoite protein R32LR antibody IgG	ELISA	in house [1]	1·9 EU/mL	Center for Vaccinology (Ghent, Belgium)
Hepatitis B virus surface antibody	CLIA	ADVIA Centaur anti–HBs2 /Siemens Healthcare	6·2 mIU/mL	GSK Clinical Laboratory Sciences (Rixensart/Wavre, Belgium)
P. falciparum circumsporozoite protein R32LR antibody IgG avidity	ELISA	in house	NA	Center for Vaccinology (Ghent, Belgium)
P. falciparum parasites	blood slide microscope reading	NA	NA	KEMRI/USAMRU–K, WRP, Malaria Diagnostics Center (Kisumu, Kenya)

IgG, immunoglobulin G; ELISA, enzyme–linked immunosorbent assay; EU, ELISA units; CLIA, chemiluminescence immunoassay; IU, international units; NA, not applicable; KEMRI/USAMRU-K, Kenya Medical Research Institute / US Army Medical Research Unit Kenya; WRP, Walter Reed Project.

References:

1. Clement F, Dewar V, Van Braeckel E, et al. Validation of an enzyme–linked immunosorbent assay for the quantification of human IgG directed against the repeat region of the circumsporozoite protein of the parasite *Plasmodium falciparum*. *Malar J* 2012; **11**:384.

Table S3. Testing available for determining the aetiology of meningitis cases and the evaluation of potential pIMDs during the study

Diagnosis of meningitis

Sample type (method)	Pathogen
Testing for aetiological pathogens	
CSF (PCR)	*H. influenzae, Streptococcus pneumoniae, Neisseria meningitidis, Salmonella enterica, Mycobacterium tuberculosis, Adenovirus, Cytomegalovirus, Enterovirus, Epstein Bar virus, Herpes simplex virus 1 & 2, HHV 6, Rabies, Mumps virus, Plasmodium spp, Toxoplasmosis*
CSF (multiplex PCR)	*Borrelia burgdorferi, Brucella spp, Coxiella burnetii, Ehrlichia spp, Leptospira spp, Rickettsia spp, Chikungunya, Crimean-Congo haemorrhagic fever virus, Dengue virus, Flavivirus genus, Hepatitis A virus, Hepatitis B virus, JC virus, Measles virus , Rift valley fever virus, Sindbis virus, Rubella virus, Varicella zoster virus, West Nile virus*
Other testing available to aid the diagnosis of meningitis (if indicated)	
Serum (PCR)	*Cytomegalovirus, Enterovirus, H. influenza, Varicella zoster virus, Toxoplasmosis*
Serum (serology)	*Beta haemolytic streptococcus, Mycoplasma pneumoniae, Streptococcus pneumoniae, Cytomegalovirus, Epstein-Barr virus, Herpes simplex virus, Measles, Mumps, Rabies, Varicella zoster virus, Cryptococcus*

Diagnosis of pIMDs*

Sample type	Test
Potential serum autoimmune tests that could be performed to aid in the diagnosis of pIMDs (if indicated)	
Serum	Anti-insulin autoantibodies (IA2), Anti-glutamic acid decarboxylase autoantibodies (anti-GAD65), Anti-Tyrosine phosphatase-like IA2 antibodies, Anti-islet cell antibodies, Anti-smooth muscle antibodies (ASMA), Anti-liver-kidney microsomal antibodies (anti-LKM), Anti-soluble liver antigens (anti-SLA), Anti-mitochondrial antibodies (AMA), Anti-nuclear antibodies (ANA), Anti-double stranded DNA (anti-dsDNA), Rheumatoid factor (RF), Anti-Glomerular Basement Membrane antibodies (anti-GBM), Anti-neutrophil cytoplasmic autoantibodies (ANCAs), Anti-streptolysin O / Anti-DNAse, Serum C3, C4 complement, Anti-cyclic citrullinated peptide antibodies (anti-CCP), Anti-skin basement membrane protein, IgA endomysial antibodies, Anticardiolipin (ELISA) IgM, IgG, Anti-beta 2 glycoprotein I, Anti-prothrombin

CSF, cerebrospinal fluid; PCR, polymerase chain reaction; pIMD, potential immune-mediated disease; ELISA, enzyme-linked immunosorbent assay; IG, immunoglobulin.

Table S4. Vaccine efficacy against clinical malaria over seven and 12 months of follow-up post-dose 3 and from D0 to M20

Time period	Population	Group	N	n	T (year)	n/T	VE (%)	95% CI	p-value
**VE against first or only episode of clinical malaria (primary case definition)**									
M2·5-M9 (7M post-dose 3)	PPS	Control	264	59	109·94	0·54			
		R012-20 + R012-14	523	51	238·78	0·21	63	46–74	<0·001
		Fx012-14	271	33	122·68	0·27	53	28–69	<0·001
M7·5-M14 (7M post-dose 3)	PPS	Control	235	80	91·79	0·87			
		Fx017-20	273	58	113·39	0·51	46	24–61	<0·001
M2·5-M14 (12M post-dose 3)	PPS	Control	265	107	176·03	0·61			
		R012-20 + R012-14	523	142	398·22	0·36	47	31–59	<0·001
		R012-20*	259	70	189·47	0·37	44	24–58	<0·001
		R012-14*	264	72	208·75	0·34	49	31–62	<0·001
		Fx012-14	271	92	204·33	0·45	35	13–51	0·003
M7·5-M19 (12M post-dose 3)	PPS	Control	236	113	142·85	0·79			
		Fx017-20	273	75	187·93	0·40	54	38–66	<0·001
**VE against first or only episode of clinical malaria (secondary case definition)**									
M2·5-M9 (7M post-dose 3)	PPS	Control	264	85	103·21	0·82			
		R012-20 + R012-14	523	95	228·54	0·42	54	38–65	<0·001
		Fx012-14	271	44	119·25	0·37	58	40–71	<0·001
M7·5-M14 (7M post-dose 3)	PPS	Control	235	109	82·79	1·32			
		Fx017-20	273	85	107·32	0·79	46	28–59	<0·001
M2·5-M14 (12M post-dose 3)	PPS	Control	265	142	158·18	0·90			
		R012-20 + R012-14	523	192	369·10	0·52	49	36–59	<0·001
		Fx012-14	271	122	191·06	0·64	39	22–52	<0·001
M7·5-M19 (12M post-dose 3)	PPS	Control	236	138	125·62	1·10			
		Fx017-20	273	103	173·31	0·59	52	37–63	<0·001
**VE against all episodes of clinical malaria (primary case definition)**									
M2·5-M9 (7M post-dose 3)	PPS	Control	264	86	121·44	0·71			
		R012-20 + R012-14	523	77	246·32	0·31	65	45–77	<0·001
		Fx012-14	271	38	127·09	0·30	59	35–74	<0·001
M7·5-M14 (7M post-dose 3)	PPS	Control	235	123	106·50	1·15			
		Fx017-20	273	87	124·54	0·70	47	26–62	<0·001
M2·5-M14 (12M post-dose 3)	PPS	Control	265	198	212·10	0·93			
		R012-20 + R012-14	523	263	436·76	0·60	44	27–57	<0·001
		Fx012-14	271	159	232·79	0·68	33	11–49	0·006
M7·5-M19 (12M post-dose 3)	PPS	Control	236	239	187·70	1·27			
		Fx017-20	273	153	218·82	0·70	54	38–66	<0·001
D0-M20	ES	Control	293	476	406·45	1·17			
		R012-20	298	341	408·64	0·83	39	23–51	<0·001
		R012-14	294	237	405·18	0·58	53	41–63	<0·001
		Fx012-14	304	287	419·43	0·68	44	30–55	<0·001
		Fx017-20	311	310	440·54	0·70	46	32–57	<0·001
**VE against all episodes of clinical malaria (secondary case definition)**									
**Overall**									
M1-M7	PPS	Control	265	118	110·97	1·06			
		Fx017-20	273	90	126·67	0·71	36	10–55	0·011
M2·5-M9 (7M post-dose 3)	PPS	Control	264	141	119·41	1·18			
		R012-20 + R012-14	523	148	243·72	0·61	57	40–70	<0·001
		Fx012-14	271	58	126·36	0·46	64	47–76	<0·001
M7·5-M14	PPS	Control	235	204	103·51	1·97			

Time period	Population	Group	N	n	T (year)	n/T	VE (%)	95% CI	p-value
(7M post-dose 3)		Fx017-20	273	157	122·02	1·29	44	26–58	<0·001
M2·5-M14 (12M post-dose 3)	PPS	Control	265	325	207·35	1·57			
		R012-20 + R012-14	523	461	429·43	1·07	44	28–56	<0·001
		Fx012-14	271	259	229·17	1·13	35	17–49	<0·001
M7·5-M19 (12M post-dose 3)	PPS	Control	236	396	181·88	2·18			
		Fx017-20	273	267	214·57	1·24	55	41–66	<0·001
D0-M14	ES	Control	293	419	267·16	1·57			
		R012-20*	298	306	269·15	1·14	37	20–51	<0·001
		R012-14*	294	273	286·62	0·95	46	31–58	<0·001
		Fx012-14	304	320	297·55	1·08	35	19–48	<0·001
		Fx017-20	311	306	294·40	1·04	39	24–52	<0·001
D0-M20	ES	Control	293	780	394·98	1·97			
		R012-20	298	619	398·16	1·55	34	20–47	<0·001
		R012-14	294	405	398·84	1·02	54	43–63	<0·001
		Fx012-14	304	460	412·85	1·11	46	34–55	<0·001
		Fx017-20	311	517	432·76	1·19	47	35–57	<0·001
**Ghana**									
M1-M7	PPS	Control	141	19	63·2	0·3			
		Fx017-20	141	23	67·35	0·34	-13	-117–41	0·704
M2·5-M9 (7M post-dose 3)	PPS	Control	140	30	68·05	0·44			
		R012-20 + R012-14	269	24	130·8	0·18	61	27–79	0·003
		Fx012-14	136	19	65·89	0·29	35	-17–63	0·149
M7·5-M14 (7M post-dose 3)	PPS	Control	131	37	64·08	0·58			
		Fx017-20	141	19	69·85	0·27	56	16–77	0·014
M2·5-M14 (12M post-dose 3)	PPS	Control	141	61	121·14	0·5			
		R012-20 + R012-14	269	62	234·61	0·26	51	24–69	0·002
		Fx012-14	136	48	121·07	0·4	23	-20–51	0·245
M7·5-M19 (12M post-dose 3)	PPS	Control	131	72	114·61	0.63			
		Fx017-20	141	33	123·18	0·27	63	36–79	0·001
D0-M14	ES	Control	147	74	150·43	0·49			
		R012-20+R012-14	304	78	303·09	0·26	52	27–68	<0·001
		Fx012-14	148	66	155·42	0·42	17	-26–45	0·379
		Fx017-20	151	48	156·48	0·31	42	8–63	0·022
D0-M20	ES	Control	147	137	221·59	0·62			
		R012-20	153	58	219·44	0·26	60	40–73	<0·001
		R012-14	151	58	220·51	0·26	59	40–72	<0·001
		Fx012-14	148	99	221·56	0·45	32	3–53	0·033
		Fx017-20	151	74	227·78	0·32	53	30–69	<0·001
**Kenya**									
M1-M7	PPS	Control	124	99	47·77	2·07			
		Fx017-20	132	67	59·32	1·13	49	23–66	0·002
M2·5-M9 (7M post-dose 3)	PPS	Control	124	111	51·36	2·16			
		R012-20 + R012-14	254	124	112·92	1·1	56	34–70	<0·001
		Fx012-14	135	39	60·47	0·64	74	57–85	<0·001
M7·5-M14 (7M post-dose 3)	PPS	Control	104	167	39·43	4·24			
		Fx017-20	132	138	52·17	2·65	41	18–57	0·002
M2·5-M14 (12M post-dose 3)	PPS	Control	124	264	86·21	3·06			
		R012-20 + R012-14	254	399	194·81	2·05	40	20–55	<0·001
		Fx012-14	135	211	108·1	1·95	39	19–54	<0·001
M7·5-M19 (12M post-dose 3)	PPS	Control	105	324	67·27	4·82			
		Fx017-20	132	234	91·39	2·56	52	35–65	<0·001

Time period	Population	Group	N	n	T (year)	n/T	VE (%)	95% CI	p-value
D0-M14	ES	Control	146	345	116·73	2·96			
		R012-20+R012-14	288	501	252·68	1·98	39	21–52	<0·001
		Fx012-14	156	254	142·12	1·79	42	25–55	<0·001
		Fx017-20	160	258	137·92	1·87	39	20–53	<0·001
D0-M20	ES	Control	146	643	173·39	3·71			
		R012-20	145	561	178·72	3·14	20	-1–37	0·066
		R012-14	143	347	178·33	1·95	51	37–62	<0·001
		Fx012-14	156	361	191·29	1·89	51	39–62	<0·001
		Fx017-20	160	443	204·99	2·16	45	30–57	<0·001

N, number of participants in each group contributing to the considered evaluation period; n, number of episodes included in each group; T (year), person years at risk; n/T, person year rate in each group; VE, vaccine efficacy; CI, confidence interval; M, month; PPS, per-protocol set; ES, exposed set; D, day.

Note: *Post-hoc analysis.
The trial was not powered to assess efficacy by country.

Table S5. Incremental vaccine efficacy of a third (fractional) dose at month 7 (Fx017-20) versus a fractional dose at month 2 (Fx012-14) over 7 and 12 months post-dose 3 (per-protocol set for efficacy)

Time period	Group	N	n	T (year)	n/T	IVE %	95% CI	p-value
**IVE against first or only episodes of clinical malaria (primary case definition)**								
7M post-dose 3	Fx012-14	271	33	122·68	0·27			
	Fx017-20	273	58	113·39	0·51	-38	-140–20	0·250
12M post-dose 3	Fx012-14	271	92	204·33	0·45			
	Fx017-20	273	75	187·93	0·40	17	-23–45	0·349
**IVE against first or only episode of clinical malaria (secondary case definition)**								
7M post-dose 3	Fx012-14	271	44	119·25	0·37			
	Fx017-20	273	85	107·32	0·79	-66	-168–-3	0·036
12M post-dose 3	Fx012-14	271	122	191·06	0·64			
	Fx017-20	273	103	173·31	0·59	10	-28–36	0·568
**IVE against all episodes of clinical malaria (primary case definition)**								
7M post-dose 3	Fx012-14	271	38	127·09	0·30			
	Fx017-20	273	87	124·54	0·70	-136	-266–-52	<0·001
12M post-dose 3	Fx012-14	271	159	232·79	0·68			
	Fx017-20	273	153	218·82	0·70	-2	-41–26	0·890
**IVE against all episodes of clinical malaria (secondary case definition)**								
7M post-dose 3	Fx012-14	271	58	126·36	0·46			
	Fx017-20	273	157	122·02	1·29	-197	-343–-99	<0·001
12M post-dose 3	Fx012-14	271	259	229·17	1·13			
	Fx017-20	273	267	214·57	1·24	-7	-43–20	0·653

IVE, incremental vaccine efficacy; M, month; N, number of participants in each group contributing to the considered evaluation period; n, number of episodes included in each group; T (year), person years at risk; n/T, person year rate in each group; CI, confidence interval.

Table S6. Parasite density at each cross-sectional visit, by group

	Group R012-20 (N=298)			Group R012-14 (N=294)			Group Fx012-14 (N=304)			Group Fx017-20 (N=311)			Control group (N=293)		
	**n**	**GM**	**Range**	**n**	**GM**	**Range**	**n**	**GM**	**Range**	**n**	**GM**	**Range**	**n**	**GM**	**Range**
M0	30	913·9	31–9086	18	1794·3	27–58564	14	1430·7	39–26253	16	1560·6	15–16428	21	3334·0	39–526070
M1	11	1361·5	79–12073	17	2970·0	77–409458	9	1227·5	113–13952	17	2211·6	67–29373	16	1255·3	51–20099
M2	12	1530·8	31–16766	13	3036·4	420–44911	8	2644·8	227–11941	16	1204·5	90–122474	11	1366·2	31–24971
M3	8	2978·4	230–28750	11	1862·8	44–22273	9	2114·5	165–7014	18	6691·3	75–431002	15	3329·5	77–489292
M4	10	1570·6	57–105000	12	5458·3	134–522763	6	1102·8	327–3367	14	3782·0	70–129976	15	2083·3	106–193875
M5	10	3468·3	67–311769	9	1810·2	210–112250	5	4561·8	127–160565	13	26766·3	117–251707	22	2637·7	159–100623
M6	12	8701·2	73–199374	11	1506·9	154–313688	8	13849·4	445–341394	17	8241·5	142–487494	18	4329·0	78–211808
M7	15	11464·4	421–244987	19	5037·0	80–342929	17	10539·0	87–231409	15	5966·2	79–333467	12	734·0	38–15055
M8	10	5919·8	61–187250	18	8018·5	152–1204419	13	3197·1	77–626628	12	3527·5	240–74498	24	3996·3	83–396485
M9	17	4805·2	38–236273	17	3826·4	83–514356	18	6751·4	79–1202126	19	7551·8	134–433770	24	4986·2	39–421248
M10	15	6388·4	190–798154	13	1296·8	55–62450	20	5599·3	60–375500	18	2321·1	71–126491	32	3125·3	55–231247
M11	21	3517·2	22–175713	14	978·9	71–116190	18	27049·4	201–781353	21	3496·0	38–337305	32	3391·4	72–171847
M12	24	8162·0	95–575109	16	3040·2	67–311087	18	4649·8	71–389776	18	3985·8	71–518459	34	5973·6	39–437436
M13	18	16607·2	112–1012917	17	12863·4	54–404846	20	8296·9	39–525190	26	13305·6	172–1252897	35	5887·6	68–639580
M14	31	2719·7	52–230217	5	671·8	71–18450	14	1622·8	38–258795	19	14344·5	45–1121049	33	3198·7	38–347338
M15	20	4312·1	95–422788	9	3982·2	39–1105175	13	4114·8	39–367423	17	1337·3	83–310805	23	2123·1	31–420936
M16	19	7499·1	71–717896	16	2233·8	27–120468	17	884·9	23–1010569	15	1117·8	38–233010	17	14816·2	216–569336
M17	26	6515·0	23–466369	24	1749·4	74–418136	18	1994·8	38–626658	15	4720·4	93–693406	29	7890·5	29–674630
M18	22	1946·8	22–147415	24	4217·9	23–482500	18	10522·4	86–777231	17	2372·9	71–183916	30	3575·6	82–156245
M19	16	1341·1	39–26974	19	5406·7	31–1147878	22	9956·9	93–433950	25	2061·9	123–284605	25	7726·2	22–348837
M20	17	1444·4	44–108167	19	4977·8	192–422973	17	15021·5	22–363060	8	1160·4	94–63640	18	9201·0	68–256003

N, total number of children in each group; n, number of children with parasite density >0 parasites/µL; GM, geometric mean of positive parasite densities (parasites/µL) for each month in each group; M, month.

Note: Range is presented as the minimum and maximum values for parasite density (parasites/µL) at each month in each group.

Table S7. Vaccine efficacy against first or only clinical episode of incident *P. falciparum* infections from D0 to M20*, overall and by country (exposed set)

Group	N	n	T (year)	n/T	VE %	95% CI	p-value
**Overall**							
Control	272	157	197·14	0·80			
R012-20	268	97	229·46	0·42	47	32–59	<0·001
R012-14	275	110	219·66	0·50	39	22–52	<0·001
Fx012-14	290	132	234·17	0·56	36	20–50	<0·001
Fx017-20	295	129	238·82	0·54	36	19–49	<0·001
**Ghana**							
Control	141	68	143·66	0·47			
R012-20	142	21	167·20	0·13	75	59–85	<0·001
R012-14	145	40	157·22	0·25	48	22–65	0·001
Fx012-14	142	45	155·13	0·29	40	13–59	0·008
Fx017-20	150	41	165·90	0·25	48	24–65	<0·001
**Kenya**							
Control	131	89	53·48	1·66			
R012-20	126	76	62·27	1·22	26	-1–45	0·058
R012-14	130	70	62·44	1·12	32	7–50	0·017
Fx012-14	148	87	79·04	1·10	34	11–51	0·007
Fx017-20	145	88	72·92	1·21	27	1–46	0·042

D, day; M, month; N, number of participants in each group contributing to the considered evaluation period; n, number of episodes included in each group; T (year), person years at risk; n/T, person year rate in each group; VE, vaccine efficacy; CI, confidence interval.

Note: *Only children with no parasitaemia (*P. falciparum* asexual parasitaemia =0 parasites/μL) at the start of follow-up period (D0) are included in this analysis. The trial was not powered to assess efficacy by country.

Table S8. Vaccine efficacy against all episodes of prevalent *P. falciparum* infections from M1 to M20, overall and by country (exposed set)

Group	N	n	T (year)	n/T	VE %	95% CI	p-value
**Overall**							
Control	293	447	405·75	1·10			
R012-20	298	317	399·84	0·79	42	22–57	<0·001
R012-14	294	281	388·02	0·72	39	18–54	<0·001
Fx012-14	304	270	408·11	0·66	45	27–58	<0·001
Fx017-20	311	332	430·74	0·77	38	18–53	<0·001
**Ghana**							
Control	147	74	221·26	0·33			
R012-20	153	27	213·11	0·13	68	31–85	0·004
R012-14	151	44	214·55	0·21	35	-25–66	0·199
Fx012-14	148	40	219·99	0·18	53	3–78	0·040
Fx017-20	151	46	224·01	0·21	53	2–77	0·043
**Kenya**							
Control	146	373	184·50	2·02			
R012-20	145	290	186·72	1·55	30	6–48	0·019
R012-14	143	237	173·46	1·37	39	17–55	0·002
Fx012-14	156	230	188·12	1·22	42	24–56	<0·001
Fx017-20	160	286	206·72	1·38	33	12–49	0·005

M, month; N, number of participants in each group contributing to the considered evaluation period; n, number of episodes included in each group; T (year), person years at risk; n/T, person year rate in each group; VE, vaccine efficacy; CI, confidence interval.

Note: The number of prevalent infections in vaccine versus Control groups was analysed by negative binomial regression. The trial was not powered to assess efficacy by country.

Table S9. Cases averted of clinical malaria (secondary case definition), by 3-month periods, from M0 to M20, overall and by country (exposed set)

	Cumulative number of cases averted per 1000 persons (95% CI)			
3-month period	**Group R012-20**	**Group R012-14**	**Group Fx012-14**	**Group Fx017-20**
**Overall**				
[0-3]	56·3 (-51·2–156·5)	121·4 (23·1–217·4)	117·2 (21·8–219·6)	73·7 (-28·2–169·7)
[0-6]	204·8 (35·9–356·2)	311·0 (164·9–457·3)	326·6 (182·9–472·4)	204·5 (37·0–353·9)
[0-9]	338·3 (76·9–575·7)	498·5 (281·2–725·3)	488·5 (269·9–711·1)	357·0 (127·1–577·2)
[0-12]	435·2 (60·1–797·1)	778·2 (447·3–1092·7)	645·2 (333·5–980·1)	610·6 (279·8–945·3)
[0-15]	526·4 (17·4–1027·5)	1060·5 (613·9–1474·6)	922·4 (515·9–1343·9)	887·3 (433·7–1321·6)
[0-18]	653·5 (4·6–1268·4)	1440·0 (890·7–1963·9)	1295·4 (775·5–1823·8)	1236·0 (673·0–1781·4)
[0–20]	700·7 (-32·6–1397·2)	1796·3 (1136·8–2414·7)	1490·3 (899·1–2113·0)	1287·8 (591·5–1928·0)
**Ghana**				
[0-3]	7·9 (-55·3–71·0)	14·5 (-45·8–74·1)	-20·0 (-80·3–43·2)	21·6 (-31·6–74·2)
[0-6]	91·6 (-0·8–180·5)	105·5 (9·6–203·4)	43·5 (-53·2–144·3)	79·1 (-15·3–165·3)
[0-9]	219·1 (92·9–353·1)	177·2 (40·2–295·3)	57·1 (-88·9–208·2)	110·3 (-30·0–252·1)
[0-12]	279·0 (110·3–447·1)	233·1 (55·1–400·4)	53·9 (-142·6–245·1)	197·2 (27·1–366·2)
[0-15]	369·6 (179·6–574·5)	318·0 (115·6–520·2)	146·5 (-73·0–359·9)	305·3 (110·4–513·8)
[0-18]	540·4 (313·3–764·9)	527·3 (280·7–758·1)	281·7 (6·3–541·0)	465·8 (194·1–724·1)
[0–20]	643·8 (363·1–952·2)	650·2 (361·6–954·0)	267·7 (-92·2–631·2)	490·7 (160·0–822·8)
**Kenya**				
[0-3]	112·4 (-85·2–305·1)	239·8 (68·1–429·2)	278·2 (113·0–461·0)	149·0 (-60·4–346·7)
[0-6]	341·7 (15·4–636·5)	546·5 (273·9–830·0)	659·3 (391·3–909·9)	377·9 (68·6–660·0)
[0-9]	494·0 (-5·5–978·0)	878·5 (448·7–1305·1)	1013·4 (613·6–1393·2)	692·6 (265·5–1115·2)
[0-12]	678·4 (-39·4–1392·5)	1488·7 (878·3–2100·8)	1444·3 (837·2–2017·6)	1233·0 (614·4–1809·4)
[0-15]	814·2 (-122·9–1764·0)	2076·2 (1315·7–2885·8)	2028·5 (1239·8–2807·0)	1806·9 (980·7–2578·5)
[0-18]	927·0 (-209·5–2058·4)	2684·2 (1799·3–3694·4)	2745·1 (1793·7–3684·1)	2494·7 (1523·9–3440·2)
[0–20]	759·4 (-674·8–2159·5)	3341·7 (2060·7–4680·9)	2879·4 (1115·8–4323·9)	2455·7 (1082·3–3769·8)

M, month; CI, confidence interval.

Note: The period M0–M20 was first divided in consecutive 3-month intervals. The number of cases averted in each of these 3-month intervals was first computed. The last interval stops at the M20 visit date. The earlier intervals are cut by 3-month intervals (30.5 days times 3). To compute the cumulated number of cases averted, the number of cases averted were summed over all the previous 3-month intervals included in the period considered, starting from day 0. The 95% CI are the equal-tail quantiles that correspond to the confidence level (2.5 to 97.5%) of the cumulative number of averted cases computed for each of the 1000 resamples with replacement (bootstrapping).

Table S10. Incidence of clinical malaria episodes by 3-month periods, from M0 to M20, overall and by country (exposed set)

3-month period	Group R012-20			Group R012-14			Group Fx012-14			Group Fx017-20			Control group		
	**n**	**T (year)**	**n/T (95% CI)**	**n**	**T (year)**	**n/T (95% CI)**	**n**	**T (year)**	**n/T (95% CI)**	**n**	**T (year)**	**n/T (95% CI)**	**n**	**T (year)**	**n/T (95% CI)**
**Overall**															
[0-3]	66	71.52	0·92 (0·74–1·16)	47	70.94	0·66 (0·51–0·87)	50	73.63	0·68 (0·52–0·88)	64	75.02	0·85 (0·68–1·07)	80	69.69	1·15 (0·94–1·41)
]3-6]	43	68.14	0·63 (0·49–0·81)	32	68.57	0·47 (0·35–0·63)	28	72.21	0·39 (0·28–0·53)	51	72.66	0·7 (0·56–0·89)	82	66.93	1·23 (1·02–1·47)
]6-9]	74	63.95	1·16 (0·92–1·46)	62	65.88	0·94 (0·73–1·21)	71	68.05	1·04 (0·82–1·32)	75	69.38	1·08 (0·86–1·36)	109	64.46	1·69 (1·4–2·05)
]9-12]	136	60.04	2·27 (1·85–2·77)	97	63.22	1·53 (1·21–1·95)	130	64.16	2·03 (1·65–2·49)	110	67.12	1·64 (1·31–2·05)	163	61.44	2·65 (2·21–3·19)
]12-15]	126	59.75	2·11 (1·72–2·58)	84	62.49	1·34 (1·05–1·72)	88	64.49	1·36 (1·07–1·74)	91	66.59	1·37 (1·08–1·74)	150	60.64	2·47 (2·05–2·98)
]15-18]	135	57.97	2·33 (1·92–2·82)	74	56.09	1·32 (1·02–1·71)	78	57.98	1·35 (1·05–1·73)	94	65.17	1·44 (1·15–1·81)	162	57.10	2·84 (2·38–3·38)
]18-20]	34	18.93	1·80 (1·38–2·34)	7	12.53	0·56 (0·31–1·00)	16	13.28	1·20 (0·82–1·77)	32	18.00	1·78 (1·35–2·34)	34	17.13	1·98 (1·52–2·59)
**Ghana**															
[0-3]	8	37.29	0·21 (0·14–0·32)	7	37.18	0·19 (0·12–0·29)	12	36.78	0·33 (0·23–0·46)	6	37.55	0·16 (0·1–0·26)	9	36.57	0·25 (0·17–0·36)
]3-6]	6	35.46	0·17 (0·10–0·27)	5	35.75	0·14 (0·08–0·24)	9	36.03	0·25 (0·17–0·37)	10	36.51	0·27 (0·19–0·40)	18	35.70	0·50 (0·38–0·67)
]6-9]	5	34.14	0·15 (0·08–0·28)	13	35.18	0·37 (0·25–0·55)	21	34.89	0·60 (0·44–0·82)	19	35.75	0·53 (0·38–0·74)	23	35.05	0·66 (0·49–0·88)
]9-12]	12	33.25	0·36 (0·24–0·54)	13	34.52	0·38 (0·25–0·56)	21	34.25	0·61 (0·45–0·84)	9	35.61	0·25 (0·16–0·41)	21	34.98	0·60 (0·44–0·82)
]12-15]	8	33.23	0·24 (0·15–0·38)	9	34.11	0·26 (0·17–0·40)	8	34.36	0·23 (0·15–0·36)	6	35.08	0·17 (0·10–0·29)	21	34.81	0·60 (0·46–0·79)
]15-18]	12	32.91	0·36 (0·24–0·55)	7	33.20	0·21 (0·12–0·36)	17	33.51	0·51 (0·36–0·72)	14	34.49	0·41 (0·28–0·60)	35	33.40	1·05 (0·82–1·34)
]18-20]	6	13.32	0·45 (0·28–0·73)	4	10.75	0·37 (0·21–0·67)	11	11.96	0·92 (0·65–1·31)	10	13.09	0·76 (0·53–1·11)	10	11.58	0·86 (0·60–1·25)
**Kenya**															
[0-3]	58	34.23	1·69 (1·29–2·23)	40	33.76	1·18 (0·85–1·65)	38	36.86	1·03 (0·74–1·45)	58	37.47	1·55 (1·18–2·03)	71	33.12	2·14 (1·67–2·74)
]3-6]	37	32.68	1·13 (0·83–1·55)	27	32.81	0·82 (0·57–1·19)	19	36.18	0·53 (0·34–0·82)	41	36.15	1·13 (0·84–1·53)	64	31.22	2·05 (1·61–2·61)
]6-9]	69	29.81	2·31 (1·77–3·03)	49	30.70	1·60 (1·16–2·19)	50	33.17	1·51 (1·10–2·07)	56	33.64	1·66 (1·24–2·24)	86	29.41	2·92 (2·30–3·72)
]9-12]	124	26.78	4·63 (3·70–5·79)	84	28.70	2·93 (2·23–3·84)	109	29.91	3·64 (2·87–4·63)	101	31.50	3·21 (2·50–4·11)	142	26.46	5·37 (4·35–6·62)
]12-15]	118	26.52	4·45 (3·54–5·59)	75	28.38	2·64 (1·99–3·52)	80	30.12	2·66 (2·01–3·50)	85	31.52	2·70 (2·06–3·53)	129	25.84	4·99 (4·01–6·21)
]15-18]	123	25.06	4·91 (3·98–6·06)	67	22.89	2·93 (2·20–3·89)	61	24.47	2·49 (1·85–3·36)	80	30.67	2·61 (2·01–3·39)	127	23.70	5·36 (4·36–6·59)
]18-20]	28	5.61	4·99 (3·57–6·98)	3	1.78	1·69 (0·61–4·71)	5	1.32	3·78 (1·71–8·37)	22	4.92	4·47 (3·06–6·54)	24	5.56	4·32 (3·00–6·21)

M, month; n, number of episodes included in each group; T (year), person years at risk; n/T, person year rate in each group; CI, confidence interval.

Note: The last interval stops at the M20 visit date. The earlier intervals are cut by 3-month intervals (30.5 days times 3). 95% CIs were computed in a post-hoc analysis using the generalized linear model with the Poisson distribution, the log as the link function, an offset (log time) and deviance as the scale with the group variable as fixed covariable (factor with the four active groups and the control group being the reference group).

Table S11. Summary of immune responses to vaccination (immunogenicity subset, per-protocol set for immunogenicity)

	Group R012-20 (N=46)	Group R012-14 (N=49)	Group Fx012-14 (N=45)	Group Fx017-20 (N=48)	Control group (N=48)
Percentage of children with anti-CS antibody concentration ≥1·9 EU/mL, % (95% CI)					
Pre-vaccination	4 (1–15)	2 (0–11)	0 (0–8)	4 (1–14)	4 (1–14)
1 month post-dose 2	100 (92–100)	100 (93–100)	100 (92–100)	98 (89–100)	9 (2–20)
1 month post-dose 3	100 (91–100)	100 (92–100)	98 (87–100)	100 (92–100)	5 (1–15)
Pre-dose 4	100 (91–100)	98 (87–100)	100 (91–100)	100 (92–100)	
1 month post-dose 4	100 (84–100)	100 (91–100)	100 (91–100)	100 (88–100)	
Anti-CS antibody GMCs, EU/mL (95% CI)					
Pre-vaccination	1 (0·9–1)	1 (0·9–1)	1 (1–1)	1 (0·9–1)	1 (0·9–1·1)
1 month post-dose 2	376·6 (294–482·4)	320·0 (248·2–412·6)	378·8 (279·3–513·9)	253·6 (182·2–353)	1·3 (0·9–1·9)
1 month post-dose 3	492·9 (383·8–633)	342·5 (275·6–425·6)	251·1 (170·6–369·6)	142·3 (113·9–177·8)	1 (0·9–1·1)
Pre-dose 4	27·7 (19·9–38·5)	26·9 (19·2–37·7)	22·6 (17·9–28·5)	33·3 (23·5–47·3)	
1 month post-dose 4	325·7 (242·4–437·7)	234·8 (184·2–299·5)	196·4 (150·4–256·7)	210·4 (145·6–304·2)	
Percentage of children seroprotected for anti-HBs antibodies (antibody concentration ≥10 mIU/mL), % (95% CI)*					
Pre-vaccination	93 (82–99)	98 (89–100)	89 (75–96)	83 (69–92)	90 (77–97)
1 month post-dose 2	100 (92–100)	100 (93–100)	100 (91–100)	100 (93–100)	91 (80–98)
1 month post-dose 3	100 (90–100)	100 (92–100)	100 (91–100)	100 (92–100)	91 (78–97)
Pre-dose 4	100 (91–100)	100 (91–100)	100 (91–100)	100 (92–100)	
1 month post-dose 4	100 (82–100)	100 (91–100)	100 (91–100)	100 (88–100)	
Anti-HBs antibody GMCs, mIU/mL (95% CI)					
Pre-vaccination	169 (103–277·3)	224·7 (142·3–354·7)	124·6 (72–215·5)	93·9 (52·9–166·8)	152·5 (93·4–249)
1 month post-dose 2	41220·9 (24137·4–70395·3)	39336·5 (26109·5–59264·5)	34301·2 (19896·6–59134·2)	29015·8 (17445·6–48259·4)	156·7 (83·6–293·7)
1 month post-dose 3	47038·1 (27915·8–79259·1)	37592·9 (27463·3–51458·8)	21641·4 (12408·6–37743·9)	53700·6 (38222·8–75446)	99·3 (57·4–171·6)
Pre-dose 4	5321·5 (3177·2–8913·0)	5700·6 (4033·4–8057·1)	4856·5 (3129·3–7537·0)	9700·1 (6732·0–13977·0)	
1 month post-dose 4	205211·9 (137112·0–307135·3)	137582·9 (108671·7–174185·8)	81612·1 (60532·7–110032·1)	76852·7 (53359·1–110690·4)	

N, maximum number of children with available results at any timepoint; CS, circumsporozoite protein; EU, enzyme-linked immunosorbent assay units; CI, confidence interval; GMC, geometric mean concentration; HBs, anti-hepatitis B surface; IU, international units.

Note: *Hepatitis B vaccination was an inclusion criterium at enrolment in the study.

Table S12. Solicited local and general symptoms over the 4-days follow-up period by dose, per study group (reactogenicity subset)

	% (95% CI)								
	Post-dose 3					Post-dose 4			
**Group**	**R012-20**	**R012-14**	**Fx012-14**	**Fx017-20**	**Control**	**R012-20**	**R012-14**	**Fx012-14**	**Fx017-20**
**N**	**46**	**48**	**44**	**45**	**49**	**41**	**44**	**42**	**43**
Erythema	0 (0–8)	2 (0–11)	0 (0–8)	0 (0–8)	0 (0–7)	0 (0–9)	2 (0–12)	2 (0–13)	0 (0–8)
Grade 3	0 (0–8)	0 (0–7)	0 (0–8)	0 (0–8)	0 (0–7)	0 (0–9)	0 (0–8)	0 (0–8)	0 (0–8)
MA	0 (0–8)	0 (0–7)	0 (0–8)	0 (0–8)	0 (0–7)	0 (0–9)	0 (0–8)	0 (0–8)	0 (0–8)
Pain	2 (0–12)	2 (0–11)	0 (0–8)	0 (0–8)	0 (0–7)	2 (0–13)	5 (1–15)	2 (0–13)	9 (3–22)
Grade 3	0 (0–8)	0 (0–7)	0 (0–8)	0 (0–8)	0 (0–7)	0 (0–9)	0 (0–8)	0 (0–8)	0 (0–8)
MA	0 (0–8)	0 (0–7)	0 (0–8)	0 (0–8)	0 (0–7)	2 (0–13)	2 (0–12)	0 (0–8)	2 (0–12)
Swelling	0 (0–8)	2 (0–11)	0 (0–8)	2 (0–12)	0 (0–7)	2 (0–13)	2 (0–12)	2 (0–13)	2 (0–12)
Grade 3	0 (0–8)	0 (0–7)	0 (0–8)	0 (0–8)	0 (0–7)	0 (0–9)	0 (0–8)	0 (0–8)	0 (0–8)
MA	0 (0–8)	2 (0–11)	0 (0–8)	0 (0–8)	0 (0–7)	2 (0–13)	0 (0–8)	0 (0–8)	0 (0–8)
Drowsiness	4 (1–15)	0 (0–7)	2 (0–12)	4 (1–15)	0 (0–7)	0 (0–9)	7 (1–19)	5 (1–16)	0 (0–8)
Grade 3	0 (0–8)	0 (0–7)	0 (0–8)	0 (0–8)	0 (0–7)	0 (0–9)	0 (0–8)	0 (0–8)	0 (0–8)
Related	2 (0–12)	0 (0–7)	2 (0–12)	2 (0–12)	0 (0–7)	0 (0–9)	0 (0–8)	0 (0–8)	0 (0–8)
Grade 3 related	0 (0–8)	0 (0–7)	0 (0–8)	0 (0–8)	0 (0–7)	0 (0–9)	0 (0–8)	0 (0–8)	0 (0–8)
MA	2 (0–12)	0 (0–7)	2 (0–12)	2 (0–12)	0 (0–7)	0 (0–9)	5 (1–15)	2 (0–13)	0 (0–8)
Irritability/fussiness	0 (0–8)	2 (0–11)	0 (0–8)	7 (1–18)	0 (0–7)	0 (0–9)	14 (5–27)	2 (0–13)	2 (0–12)
Grade 3	0 (0–8)	0 (0–7)	0 (0–8)	0 (0–8)	0 (0–7)	0 (0–9)	0 (0–8)	0 (0–8)	0 (0–8)
Related	0 (0–8)	0 (0–7)	0 (0–8)	4 (1–15)	0 (0–7)	0 (0–9)	7 (1–19)	0 (0–8)	0 (0–8)
Grade 3 related	0 (0–8)	0 (0–7)	0 (0–8)	0 (0–8)	0 (0–7)	0 (0–9)	0 (0–8)	0 (0–8)	0 (0–8)
MA	0 (0–8)	2 (0–11)	0 (0–8)	4 (1–15)	0 (0–7)	0 (0–9)	11 (4–25)	0 (0–8)	0 (0–8)
Loss of appetite	0 (0–8)	2 (0–11)	0 (0–8)	2 (0–12)	0 (0–7)	5 (1–17)	11 (4–25)	7 (1–19)	0 (0–8)
Grade 3	0 (0–8)	0 (0–7)	0 (0–8)	0 (0–8)	0 (0–7)	0 (0–9)	0 (0–8)	0 (0–8)	0 (0–8)
Related	0 (0–8)	0 (0–7)	0 (0–8)	0 (0–8)	0 (0–7)	2 (0–13)	2 (0–12)	2 (0–13)	0 (0–8)
Grade 3 related	0 (0–8)	0 (0–7)	0 (0–8)	0 (0–8)	0 (0–7)	0 (0–9)	0 (0–8)	0 (0–8)	0 (0–8)
MA	0 (0–8)	2 (0–11)	0 (0–8)	2 (0–12)	0 (0–7)	5 (1–17)	9 (3–22)	5 (1–16)	0 (0–8)
Fever	26 (14–41)	17 (7–30)	9 (3–22)	11 (4–24)	2 (0–11)	12 (4–26)	25 (13–40)	14 (5–29)	5 (1–16)
Grade 3	4 (1–15)	0 (0–7)	0 (0–8)	0 (0–8)	0 (0–7)	0 (0–9)	2 (0–12)	2 (0–13)	0 (0–8)
Related	20 (9–34)	15 (6–28)	9 (3–22)	2 (0–12)	0 (0–7)	10 (3–23)	11 (4–25)	5 (1–18)	5 (1–16)
Grade 3 related	4 (1–15)	0 (0–7)	0 (0–8)	0 (0–8)	0 (0–7)	0 (0–9)	2 (0–12)	0 (0–8)	0 (0–8)
MA	11 (4–24)	4 (1–14)	5 (1–15)	4 (1–15)	2 (0–11)	7 (2–20)	18 (8–33)	7 (1–19)	2 (0–12)

%, percentage of doses followed by at least one type of symptom; CI, confidence interval; N, number of doses administered, MA, medically attended·

Note: The analysis included all children within the reactogenicity subset who had safety data. Grade 3 adverse events were defined as erythema/swelling >20 mm, crying when limb is moved (pain), not eating at all (loss of appetite), preventing normal everyday activities (drowsiness, irritability/fussiness), temperature >39·0°C (fever).

Table S13. Summary of unsolicited adverse events, serious adverse events and adverse events of special interest reported from D0 to M21 (exposed set)

	% (95% CI)				
**Group**	**R012-20**	**R012-14**	**Fx012-14**	**Fx017-20**	**Control**
**N**	**298**	**294**	**304**	**311**	**293**
At least one unsolicited AE, within 30 days of any of the three first vaccination doses excluding malaria (post-hoc analysis)					
Any unsolicited AE	74·5 (69·2–79·3)	74·5 (69·1–79·4)	78 (72·9–82·5)	74·9 (69·7–79·6)	80·2 (75·2–84·6)
Grade 3	2·3 (0·9–4·8)	1·0 (0·2–3·0)	2·3 (0·9–4·7)	3·2 (1·6–5·8)	2·4 (1·0–4·9)
Related	2·7 (1·2–5·2)	2·7 (1·2–5·3)	2·3 (0·9–4·7)	2·9 (1·3–5·4)	2·0 (0·8–4·4)
Grade 3, related	0·3 (0·0–1·9)	0·3 (0·0–1·9)	0·0 (0·0–1·2)	0·3 (0·0–1·8)	0·0 (0·0–1·3)
At least one unsolicited AE, within 30 days of any of the three first vaccination doses including malaria					
Any unsolicited AE	76·8 (71·6–81·5)	78·6 (73·4–83·1)	82·6 (77·8–86·7)	79·7 (74·8–84·1)	81·2 (76·3–85·5)
Grade 3	3·4 (1·6–6·1)	2·0 (0·8–4·4)	2·6 (1·1–5·1)	4·2 (2·2–7·0)	2·7 (1·2–5·3)
Related	5·4 (3·1–8·6)	7·1 (4·5–10·7)	3·0 (1·4–5·5)	4·2 (2·2–7·0)	2·0 (0·8–4·4)
Grade 3, related	1·0 (0·2–2·9)	0·3 (0·0–1·9)	0·0 (0·0–1·2)	0·6 (0·1–2·3)	0·0 (0·0–1·3)
SAEs, excluding malaria (post-hoc analysis)					
Any SAE	12·8 (9·2–17·1)	12·2 (8·7–16·5)	11·8 (8·4–16)	16·1 (12·2–20·6)	16·7 (12·6–21·5)
Related	1·0 (0·2–2·9)	0·0 (0·0–1·2)	0·0 (0·0–1·2)	0·6 (0·1–2·3)	0·0 (0·0–1·3)
Fatal SAE	0·3 (0·0–1·9)	0·3 (0·0–1·9)	0·0 (0·0–1·2)	0·6 (0·1–2·3)	0·0 (0·0–1·3)
SAEs, including malaria					
Any SAE	16·1 (12·1–20·8)	15·3 (11·4–19·9)	15·5 (11·6–20·0)	19·9 (15·6–24·8)	24·2 (19·4–29·6)
Related	1·0 (0·2–2·9)	0·0 (0·0–1·2)	0·0 (0·0–1·2)	0·6 (0·1–2·3)	0·0 (0·0–1·3)
Leading to withdrawal	0·3 (0·0–1·9)	0·3 (0·0–1·9)	0·0 (0·0–1·2)	0·6 (0·1–2·3)	0·0 (0·0–1·3)
Fatal SAE	0·3 (0·0–1·9)	0·3 (0·0–1·9)	0·0 (0·0–1·2)	0·6 (0·1–2·3)	0·0 (0·0–1·3)
Malaria**	29·9 (24·7–35·4)	29·3 (24·1–34·8)	27·6 (22·7–33·0)	32·2 (27–37·7)	34·5 (29·0–40·2)
AEs of special interest					
At least one meningitis	0·3 (0·0–1·9)	0·0 (0·0–1·2)	0·3 (0·0–1·8)	0·6 (0·1–2·3)	0·7 (0·1–2·4)
Viral	0·3 (0·0–1·9)	0·0 (0·0–1·2)	0·3 (0·0–1·8)	0·3 (0·0–1·8)	0·7 (0·1–2·4)
Enteroviral	0·0 (0·0–1·2)	0·0 (0·0–1·2)	0·0 (0·0–1·2)	0·3 (0·0–1·8)	0·0 (0·0–1·3)
At least one seizure*	3·0 (1·4–5·7)	1·0 (0·2–3·0)	1·6 (0·5–3·8)	3·9 (2·0–6·6)	3·8 (1·9–6·6)
At least one pIMD	0·0 (0·0–1·2)	0·3 (0·0–1·9)	0·0 (0·0–1·2)	0·0 (0·0–1·2)	0·0 (0·0–1·3)
At least one severe malaria case or cerebral malaria	4·4 (2·3–7·3	4·8 (2·6–7·9)	4·9 (2·8–8·0)	6·4 (4·0–9·8)	10·9 (7·6–15·1)
At least one severe malaria	4·4 (2·3–7·3)	4·8 (2·6–7·9)–	4·9 (2·8–8·0)–	6·4 (4·0–9·8)–	10·6 (7·3–14·7)
At least one cerebral malaria	0·0 (0·0–1·2)	0·0 (0·0–1·2)–	0·0 (0·0–1·2)–	0·0 (0·0–1·2)–	0·3 (0·0–1·9)

D, day; M, month; %, percentage of children with at least one AE; N, number of vaccinated children in each group; CI, confidence interval; AE, adverse event; SAE, serious AE; pIMD, potential immune-mediated disease.

Note: The analysis included all children in the exposed set who had safety data.

*Reported within 30 days post-vaccination; ** including *P. falciparum* infection, cerebral malaria and malaria.
